# Data Driven Investigation of Bispectral Index Algorithm

**DOI:** 10.1038/s41598-019-50391-x

**Published:** 2019-09-24

**Authors:** Hyung-Chul Lee, Ho-Geol Ryu, Yoonsang Park, Soo Bin Yoon, Seong Mi Yang, Hye-Won Oh, Chul-Woo Jung

**Affiliations:** Department of Anesthesiology and Pain Medicine, Seoul National University College of Medicine, Seoul National University Hospital, Seoul, Republic of Korea

**Keywords:** Health care, Health care, Health care, Medical research, Medical research

## Abstract

Bispectral index (BIS), a useful marker of anaesthetic depth, is calculated by a statistical multivariate model using nonlinear functions of electroencephalography-based subparameters. However, only a portion of the proprietary algorithm has been identified. We investigated the BIS algorithm using clinical big data and machine learning techniques. Retrospective data from 5,427 patients who underwent BIS monitoring during general anaesthesia were used, of which 80% and 20% were used as training datasets and test datasets, respectively. A histogram of data points was plotted to define five BIS ranges representing the depth of anaesthesia. Decision tree analysis was performed to determine the electroencephalography subparameters and their thresholds for classifying five BIS ranges. Random sample consensus regression analyses were performed using the subparameters to derive multiple linear regression models of BIS calculation in five BIS ranges. The performance of the decision tree and regression models was externally validated with positive predictive value and median absolute error, respectively. A four-level depth decision tree was built with four subparameters such as burst suppression ratio, power of electromyogram, 95% spectral edge frequency, and relative beta ratio. Positive predictive values were 100%, 80%, 80%, 85% and 89% in the order of increasing BIS in the five BIS ranges. The average of median absolute errors of regression models was 4.1 as BIS value. A data driven BIS calculation algorithm using multiple electroencephalography subparameters with different weights depending on BIS ranges has been proposed. The results may help the anaesthesiologists interpret the erroneous BIS values observed during clinical practice.

## Introduction

Electroencephalography (EEG)-based monitoring of the depth of anesthesia is frequently used to detect intraoperative awareness and prevent unnecessary deep anaesthesia during general anaesthesia^1^. The bispectral index (BIS) is the most widely used measure among the various anaesthetic depth indices because of the close relationship between the BIS values and depth of anaesthesia^2^. However, the BIS may fail to reflect the level of anaesthesia under certain circumstances including external artifacts such as electromyography (EMG)^3–5^; use of medical devices such as pacemaker^6^, forced air warmer^7^, endoscopic devices^8^, electrocardiogram^9^ and cardiopulmonary bypass machine^10^; drugs such as ephedrine^11^, ketamine^12^, and etomidate^13^; clinical conditions such as cardiac arrest^14^, cerebral ischemia^15^, hypothermia^16^ and hypoglycemia^17^.

The BIS values are known to be calculated from four EEG subparameters, burst suppression ratio (BSR), QUAZI suppression index, relative beta ratio (RBR), and SyncFastSlow (SFS), using multiple regression equations with different weights according to the depth of anaesthesia^18,19^. Understanding the BIS algorithm is essential for proper diagnosis and management of abnormal BIS values that are not related to the anaesthetic state itself. However, the BIS calculation method is a proprietary algorithm: the manufacturer has not disclosed the exact calculation method of the QUAZI suppression index, the selection criteria of different regression equations, and the weights of subparameters^20,21^.

In this study, we explored the algorithm of BIS by applying decision tree analysis, a machine learning technique, to clinical big data of BIS collected during daily anaesthesia practice. The purpose of this study was to better understand the BIS algorithm by investigating the calculation process of the BIS values from the EEG subparameters.

## Results

Among the 6,388 patients included in the registry, 5,543 had undergone BIS monitoring. The number of cases with BIS recording for >30 min were 5,427. The total number of data points with SQI > 90% was 31,372,258. The training dataset included 4,342 cases (80%) and 24,881,109 data points, and the test dataset included 1,085 cases (20%) and 6,491,149 data points. The general characteristics of the patients are summarized in Table 1.

**Table 1:** Dataset characteristics.

	All (n = 5,427)	Training (n = 4,342)	Test (n = 1,085)	*P*-value
Age (years)	59 (49–69)	59 (49–68)	59 (49–69)	0.256
Sex (male/female)	2,708/2,719	2,172/2,170	536/549	0.745
Height (cm)	162 (156–168)	162 (156–169)	162 (156–168)	0.492
Weight (kg)	61 (53–69)	61 (54–69)	60 (53–69)	0.129
Anaesthesia Type				0.713
Total intravenous	2,558 (47%)	2,052 (47%)	506 (47%)	
Volatile	943 (17%)	760 (17%)	183 (17%)	
Balanced	1,930 (36%)	1,533 (35%)	397 (37%)	
Surgery duration (min)	120 (71–200)	120 (65–195)	125 (71–200)	0.113
Anaesthesia duration (min)	165 (105–250)	165 (100–245)	170 (105–250)	0.106

Data are expressed as number (%) or median (interquartile range).

Dataset comparison between the training and test datasets were performed using chi-square test or Student t-test.

### Determination of BIS ranges

**Figure 1:**
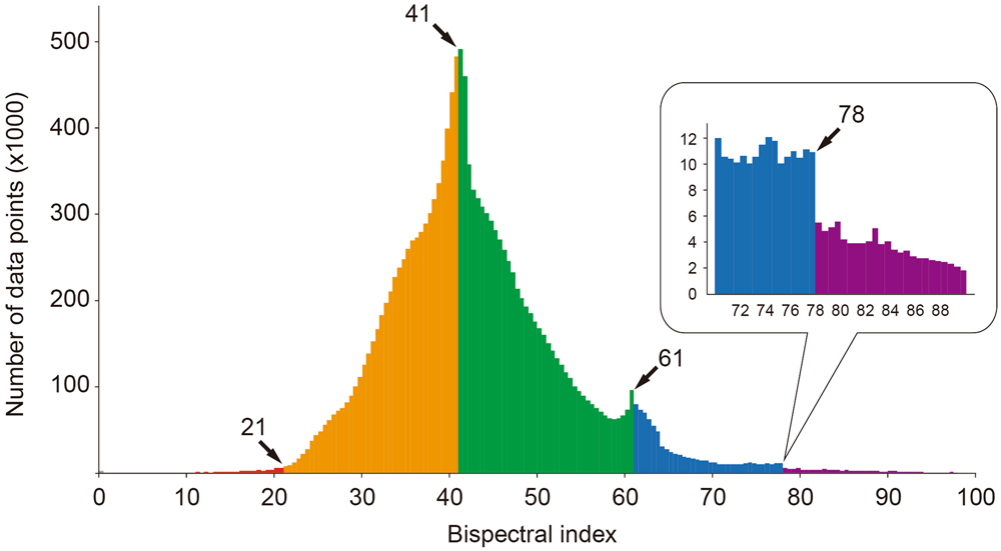
Histogram of bispectral index. The histogram from the entire dataset shows unusual data distribution at the bispectral index values 41, 61 and 78.

The median (interquartile range) BIS of data points was 42 (37–48). In the histogram of the BIS values, sudden changes in the frequency were observed at BIS values of 41, 61, and 78 (Fig. 1). By adding BIS 21, a known boundary value^22^, to these three values, five ranges of BIS were distinguished by boundary values of 21, 41, 61, and 78: 0 ≤ BIS ≤ 21, 21 < BIS ≤ 41, 41 < BIS ≤ 61, 61 < BIS ≤ 78, and 78 < BIS ≤ 98.

### Determination of BIS ranges by EEG subparameters

**Figure 2:**
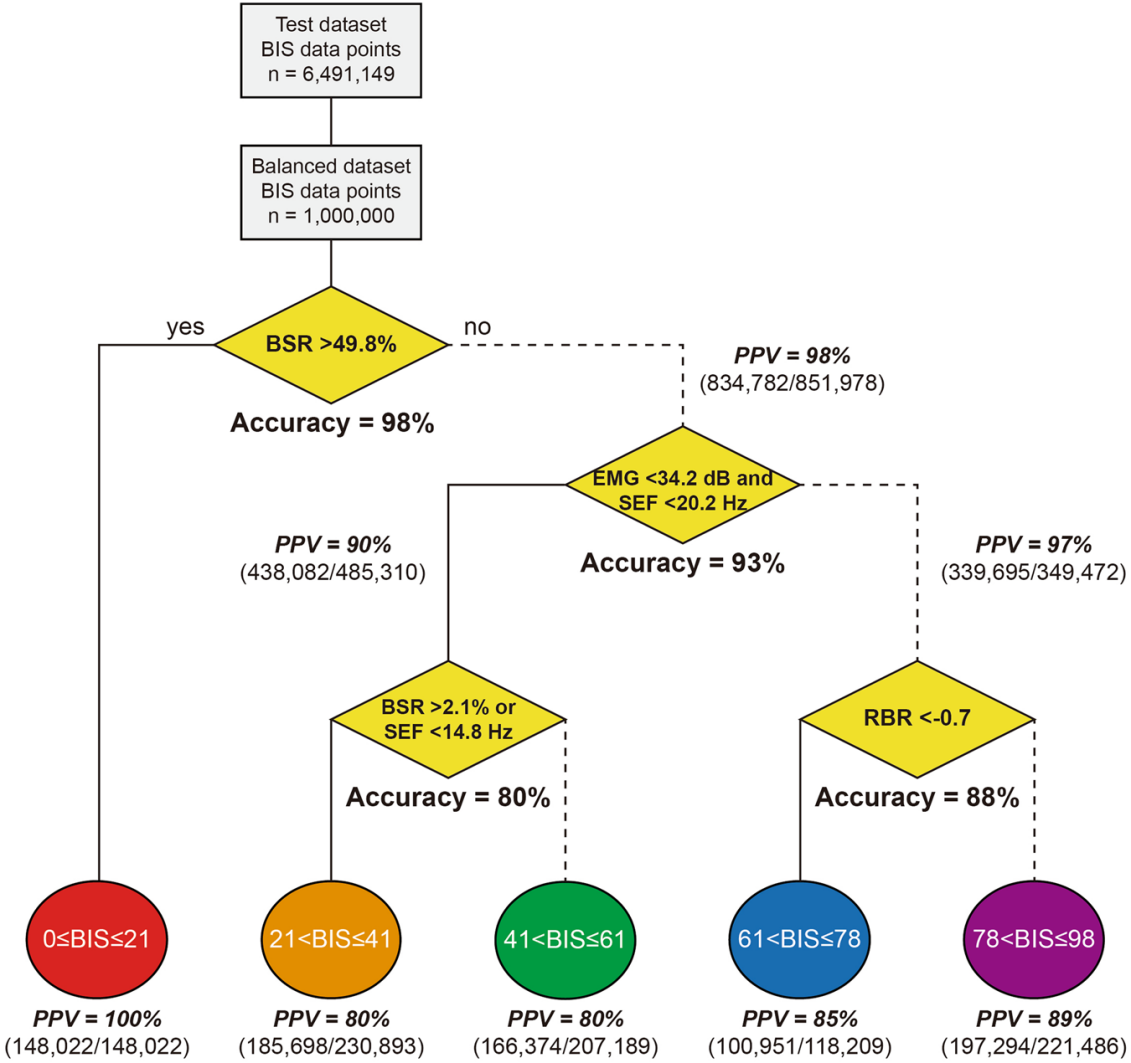
Decision tree analysis and performance evaluation. The decision tree developed with the balanced training dataset indicates that BSR, EMG, SEF, and RBR are classifiers to determine 5 different BIS ranges. The accuracy and positive predictive values calculated in the balanced test dataset are represented in the decision and leaf nodes. Abbreviations: BIS = bispectral index, BSR = burst suppression ratio, EMG = power of electromyography, PPV = positive predictive value, RBR = relative beta ratio, SEF = 95% spectral edge frequency.

Decision tree analyses revealed that the tree model was so robust that the 10 trees have the same structure and similar threshold values. Therefore, the final tree was determined to have the unique classifiers with the median values of thresholds (Fig. 2). The classifier of the root node is the BSR, and if BSR > 49.8%, the input is specified in the BIS 0–21 range. In the second level node, when EMG < 34.2 dB and SEF < 20.2 Hz are satisfied at the same time, the input is classified as BIS 21–61. Otherwise it is classified as BIS 61–98. The third decision node classifies the input as BIS 21–41 if either BSR > 2.1% or SEF < 14.8 Hz is satisfied. Otherwise it is classified as BIS 41–61. At the last decision node, RBR <−0.7 was used as the classifier of BIS 61–78 and BIS 78–98.

Figure 2 also shows the external validation results using the balanced test dataset, as well as the final decision tree model. The accuracy of the decision nodes was 98%, 93%, 80%, and 88% for the 21, 61, 41, and 78 boundaries, respectively, and the overall accuracy was 92%. Positive predictive values for each BIS range were 100%, 80%, 80%, 85%, and 89%, respectively, in the increasing direction of BIS, and the overall positive predictive value was 86%.

### Regression models

**Figure 3:**
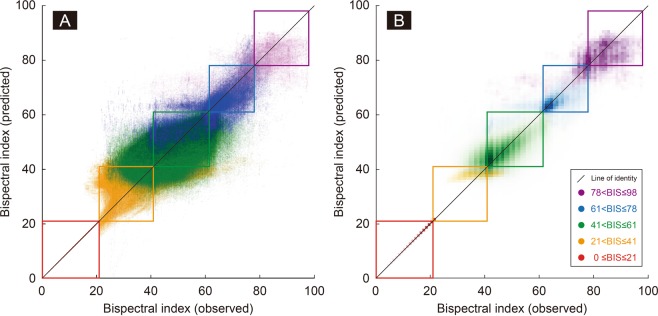
Observed *vs* model-predicted bispectral index. The scatter plot (**A**) and heat map chart (**B**) represent the agreement between the observed and the model-predicted bispectral index values in the test dataset. The darkest colors in the heat map chart indicate the points where the maximum number of data pairs in each bispectral index range is located. The majority of data points are located along the line of identity (median absolute error = 4.1 as bispectral index value) and within the designated bispectral index ranges (average positive predictive value = 89%; presented in the Fig. 2). However, in the range of bispectral index 21–41, the data pairs seem to deviate significantly from the line of identity, presumably due to the failure to include the QUAZI suppression index in the model.

Table 2 shows the multiple linear regression models with different weights for the four EEG subparameters according to the estimated BIS ranges. The right side of the table shows the median errors and the median absolute errors when the regression models were externally validated in the test dataset. The median absolute error of each BIS range was 0.0, 4.1, 4.0, 4.0, 4.6 in the order of increasing BIS value, and 4.1 for the entire range. The relationship between the observed BIS and model-predicted BIS in a balanced test dataset is represented as a scatter plot (Fig. 3A) and a heat map chart (Fig. 3B). The percentage of points of the same color contained in the color boxes in each range is high, which is consistent with the high positive predictive value of each range, shown in Fig. 2. In addition, the data points are appropriately distributed along the line of identity across the entire range, visualizing the low errors in Table 2. However, the data pairs seem to deviate significantly from the line of identity in the range of BIS 21–41, indicating that the classification and/or regression may not be appropriate for this BIS range.

**Table 2:** Multiple linear regression models to calculate bispectral index in five different BIS ranges.

BIS range	Subparameters and coefficients				Intercept	Median error	Median absolute error
	BSR	SEF	RBR	EMG			
0 ≤ BIS ≤ 21	−0.42	0.00	0.01	0.00	42.1	0.0	0.0
21 < BIS ≤ 41	−0.42	0.91	3.06	0.04	29.9	−0.6	4.1
41 < BIS ≤ 61	−3.01	3.84	−8.70	0.96	−57.6	0.2	4.0
61 < BIS ≤ 78	−1.43	2.55	4.26	0.41	5.3	0.0	4.1
78 < BIS ≤ 98	−1.97	0.88	7.89	−0.07	65.2	−0.3	4.7

The regression models are applied after determining specific BIS ranges with decision tree classifiers. The overall median absolute error in the test dataset is 4.1 as BIS value.

Abbreviation: BIS = bispectral index, BSR = burst suppression ratio, EMG = power of electromyography, RBR = relative beta ratio, SEF = 95% spectral edge frequency.

**Figure 4:**
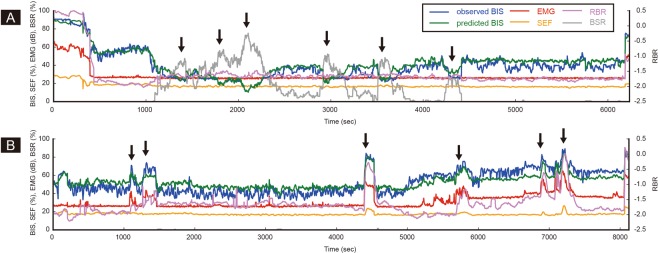
Typical examples of unusual bispectral index values during surgery. Two typical cases where BIS values are affected by specific electroencephalographic subparameters are presented. (**A**) The BIS is mainly determined by the level of BSR (arrow). (**B**) Sudden increase of EMG causes an unexpected peak of the BIS (arrow). In both cases, our model well estimates the measured BIS values. Abbreviations: BIS = bispectral index, BSR = burst suppression ratio, EMG = power of electromyography, RBR = relative beta ratio, SEF = 95% spectral edge frequency.

Figure 4 shows typical cases where the BIS values are affected by changes in the four EEG subparameters, such as BSR, EMG, SEF, and RBR. Figure 4A shows how the BIS value changes in proportion to the change in BSR in the range below BIS 60. Figure 4B shows that a sudden increase in EMG during surgery can increase the BIS value beyond 60, regardless of the anaesthetic depth. However, minimum discrepancy between the observed and the predicted BIS values proves that our model works well. Supplementary Fig. 1 presents line plots showing BSR, SEF, RBR, EMG, measured BIS, and model predicted BIS values over time in a total of randomly selected 100 cases.

## Discussion

In the current study, BIS algorithm was investigated using BIS big data and machine learning technique. Our model uses a decision tree to sequentially verify the values of BSR, EMG, SEF and RBR, and classify them into five BIS ranges. Thereafter, regression equations with different coefficients are used for each BIS range to finally calculate the BIS value. The model predicted five BIS ranges with an average accuracy of 86%, and BIS values with an average absolute error of 4.1 in external validation,

The first BIS algorithm (version 1.0) in 1992 predicted the occurrence of movement during incision through multivariate regression model using various EEG subparameters. The calculated BIS values were anaesthetic agent-specific and proportional to the anaesthetic dose^23^. Several improvements have been made to the algorithm, such as removal of EEG artifacts and adjustment of coefficients. The version 4.1 algorithm developed in 2004 is currently in use^24^. However, the only available information regarding the company’s proprietary algorithm is that four EEG subparameters are adjusted to have different weights according to the anaesthestic depth^18^. Our assumption for searching for an algorithm from data is that the anaesthetic depth or the BIS range is first determined by specific values of EEG subparameters, and afterwards a unique regression equation for that range is applied to the EEG subparameters to calculate the BIS value.

In the prospective development of the original BIS algorithm, a specific state of anaesthesia was first defined. Then a regression equation was made from the EEG subparameters of the state. However, classification of the BIS ranges was the first step of our study, since our data has no clinical information about the patients’ anaesthestic state. Therefore, we sought five different BIS ranges that represent the corresponding anaesthetic states. We assumed that the frequency of the samples would change rapidly at the points where data discontinuity occurred due to the switching of the regression equations of the BIS. The sought boundaries 41, 61, and 78 are very similar to those found in the literature, 40, 60, and 80. Therefore, the five BIS ranges, 0–21, 21–41, 41–61, 61–78, and 78–98, correspond to the five different anaesthetic states, isoelectricity, profound anaesthesia, adequate anaesthesia, sedation, and awake, respectively.

In our decision tree analysis, four decision nodes are sequentially classified without loop or remerge. The first decision node splits the BIS ≤ 21 and BIS > 21 ranges based on the BSR value of 49.8. As a result, the decision tree model shows a 100% prediction rate for the BIS ≤ 21 range, and the error of the regression model for this range is zero. The regression equation for this range, BIS = 42.1 − 0.42 × BSR + 0.01 × RBR (BSR > 49.8), is very similar to the two previous data driven results: BIS = 50 − BSR/20 (BSR ≥ 40)^25^; BIS = 44.1 − BSR/2.25 (BSR > 40)^22^.

The second decision node operates on the BIS 61, a boundary of general anaesthesia, and EMG and SEF were chosen as classifiers. It is notable that increase of EMG power was a classifier of the BIS ranges. Previous reports of an erroneous increase in BIS were related with an unexpected EMG pattern for conventional general anaesthesia, such as muscle relaxation in an awake patient^3,5^, or reversal of muscle relaxation in anaesthetized patients^26–28^; and high frequency band electrical noise^4,6–8,10^. Our results and previous reports suggest that the original BIS algorithm needs to be more improved to properly measure anaesthetic depth without being affected by high frequency signals including EMG^29^.

The EMG values in the range of BIS 21–61 were almost constant. Thus the BIS values seem to be closely related to the SEF values as shown in the regression coefficients in Table 2. In the original BIS algorithm, SFS calculated by bispectral analysis is known to be a key parameter for the calculation of BIS values. However, SFS, defined as log_10_(B_40–47Hz_/B_0.5–47Hz_), sometimes fails to represent the degree of phase coupling because the bispectral band is affected by the EMG signal. In addition, it is questionable whether SFS is used appropriately in calculating BIS values, because (1) SFS calculation based on bispectral analysis is very complex and requires a lot of computing power, and (2) BIS monitor does not output SFS value^30^. On the other hand, the SEF calculated at 0–30 Hz frequency is less affected by EMG than BIS^31^. In previous studies, the correlation of SEF with anaesthetic concentration and BIS value was superior to that of SFS, and SEF was reported to be a better indicator of actual anaesthetic depth than SFS^21,32^. In our analysis, simultaneous application of SEF and EMG not only better distinguishes BIS ranges, but also better responds to changes in BIS values with depth of anaesthesia than SFS alone.

At the third decision node, SEF 14.8 Hz or BSR 2.1% was selected as the classifier of the BIS 41 boundary to distinguish between profound anaesthesia and adequate anaesthesia. The threshold values of the classifier and the coefficients of the two regression equations shown seem to reflect two different levels of general anaesthesia appropriately. However, the accuracy of this decision node is 80%, which is the lowest among all the accuracy. This is probably due to the fact that the QUAZI suppression index, a presumed EEG subparameter for the BIS calculation in the profound anaesthetic state, was not used in our algorithm. However, since the specific calculation formula of the QUAZI suppression index is not known, none of the studies so far have been able to provide accurate analysis using the QUAZI suppression index.

In the fourth decision node, RBR is chosen as a classifier. Morimoto and colleagues analyzed the data obtained from the BIS monitor and derived an RBR formula: BIS = 20 × RBR + 95 (BIS 60–100; *R* = 0.9, *P* < 0.01)^21^. Our model also shows that BIS values are highly dependent on RBR in the range of BIS > 61. The accuracy of the RBR classifier at the BIS 78 boundary is as high as 88%, and the coefficient values of RBR in the BIS 61–98 range are relatively large. On the other hand, since RBR is defined as log_10_(P_30–47Hz_/P_11–20Hz_), BIS and RBR show positive and negative correlations in BIS > 60 and BIS < 60, respectively. In our regression model, the RBR changes to a negative regression coefficient at the boundary of BIS 61.

Our study has several limitations. First, it is possible that the BIS output from the monitor is a smoothed value from the time series BIS values, rather than the primary calculated value from subparameters. Smoothed values can cause errors in classifiers and regression equations in situations where the BIS value changes rapidly. Our model can be less accurate especially during dynamic phases such as induction and emergence periods. Second, in the EEG analysis, we processed only channel 1 EEG without excluding noisy epochs, which may be different from the signal processing in the original BIS algorithm. However, only data with SQI > 90% (validated data from the original BIS algorithm) were included to ensure the accuracy of our BIS algorithm. Third, our algorithm may have practical problems with improper inputs due to artifacts. However, artifact issues mostly relate to the calculation of the subparameters themselves, not the relationship of the subparameters, and thus is beyond the scope of the current study. Forth, in algorithms that use different regression equations for each BIS range, a rule is required to determine when the result of the regression equation falls within a different BIS range. In the histogram, excessive data points are visible at the boundaries of 41 and 61, which can increase errors in the modeling of the decision tree and/or regression equation. We tried to minimize the error due to the accumulated BIS value at the boundary by using a balanced dataset. Finally, it should be emphasized that our data driven model cannot be used to directly modify BIS results or to make an assessment of the patient’s actual anaesthesia states. However, our model may be useful for identifying erroneous BIS values. For instance, the SEF and EMG setting such as SEF < 20.2 Hz and EMG > 34.2 dB is useful to exclude intraoperative awareness even with high BIS values after recovery of EMG activity during surgery.

In conclusion, we were able to generate a model to calculate the BIS value by classifying BIS ranges from EEG subparameters and applying different multiple regression equations with different weights of subparameters for each BIS range. The classifiers and regression coefficients proposed in this study will not only help understand the changes in BIS values observed in clinical practice, but also help to interpret the unusual BIS values.

## Methods

### Dataset

Data were retrieved from the VitalDB, an open data repository of intraoperative vital signs (http://vitaldb.net/data-bank, accessed Feb 21, 2018). The data were collected from 10 operating rooms of Seoul National University Hospital from Jun 2016 to Aug 2017, using authors’ own developed data-recording software (Vital Recorder 1.7.4; https://vitaldb.net/vital-recorder, accessed Feb 21, 2018)^33^. The collection and public release of the VitalDB dataset has been approved by the institutional review board of Seoul National University Hospital (H-1408-101-605), and the construction of data repository has been also registered at publicly accessible clinical trial registration site (ClinicalTrial.gov, NCT02914444). Our institutional review board judged our current retrospective study as exempt from review due to the use of deidentified public dataset.

The dataset includes 6,388 patients who received surgical procedures under general and regional anaesthesia. Of the 6,388 cases, 80% were assigned to the training group and 20% were assigned to the test group. The training dataset was used for model training, and the test dataset was used for external validation of the model.

### Preparation of EEG subparameters

The EEG was captured from the surgical patients using the BIS Vista monitor (BISx revision 1.15, BIS engine 4.1; Medtronic, Minneapolis, MN, USA) and BIS Quatro sensor. The high-fidelity BIS data includes BIS value, signal quality index (SQI), 95% spectral edge frequency (SEF), BSR, power of electromyogram (EMG) in 1 sec interval, and two channels of 128 Hz EEG waves. The spectral and bispectral smoothing rates were set to default values such as 30 and 15 sec, respectively. In our pilot study, we observed a problem of poor model accuracy when all cases were included, possibly due to data smoothing delays during the dynamic phase. Cases with BIS recording for more than 30 min were selected to reduce the impact of dynamic phases, and data points with SQI > 90% in each case were included in the analysis.

Five subparameters such as BSR, SEF, EMG, RBR and SFS were used for model training. In the BIS monitor, the BSR value is calculated as a fraction of the segment with a signal amplitude of less than 5 μV for 60 sec^18,21^. The SEF is defined as the frequency below which 95% of the total power lies, and is in the 0.5–30.0 Hz range of EEG^32^. The EMG in the BIS monitor is defined as the absolute power in the 70–110 Hz range of EEG^3,24^. These three subparameters can be directly captured from the serial port of the BIS monitor. However, RBR and SFS were computed separately from the EEG waveform analysis because the BIS monitor does not output these values. First, the channel 1 EEG waveform was detrended using the Savitzky Golay filter^34^. The waveform was then divided into 2-sec epochs with 75% overlap, and the Fast Fourier transformation was performed after applying the Blackman window for each epoch. The power spectral values were calculated from 57 epochs made from the previous 30 sec EEG, and the bispectral values were calculated from 27 epochs made from the 15 sec EEG, according to the smoothing rate setting of the BIS monitor. From these, RBR and SFS were calculated as follows^18,20,35^.

$${\rm{RBR}}={\log }_{10}({{\rm{P}}}_{30\mbox{--}47{\rm{Hz}}}/{{\rm{P}}}_{11\mbox{--}20{\rm{Hz}}})$$

$${\rm{SFS}}={\log }_{10}({{\rm{B}}}_{0.5-47{\rm{Hz}}}/{{\rm{B}}}_{40-47{\rm{Hz}}}),$$

where P and B mean the spectral and bispectral powers in EEG band, respectively.

### Decision tree analysis

The original BIS algorithm seems to select the appropriate regression equation after determining the depth of anaesthesia from the specific reference values of EEG subparameters^18,19,21,22,25^. To investigate the BIS algorithm from our dataset, we first performed the decision tree analysis to find the EEG subparameters and their thresholds that determine the level of anaesthetic depth, and the regression analysis to estimate the different regression equations for each anaesthetic level thereafter.

The decision tree analysis is a type of supervised machine learning that requires definite and correct outputs for given inputs. The outputs were the BIS ranges that correspond to various levels of anaesthetic depths. However, the exact BIS boundary values to determine the BIS ranges are unknown. A histogram of the entire BIS values was plotted to find the BIS boundaries that would be represented as discontinuities in the distribution due to conversion to a different regression equation.

Then, a decision tree analysis was performed using EEG subparameters such as BSR, SEF, RBR, and EMG as inputs, and BIS ranges as outputs. However, inadequate learning due to the imbalanced dataset may occur since most of the BIS values are located at 40 to 60 in this intraoperative dataset^36^. Therefore, a balanced dataset of 100,000 samples, in which about 1000 samples were extracted from each value of the BIS 0–98, was used for training and external validation of the decision tree model. In addition, an ensemble method was used to prevent overfitting of the model due to data selection bias. A new balanced dataset was created for each analysis, and a total of 10 decision tree analyses were performed. Each decision tree analysis was performed according to an optimized Classification and Regression Trees (CART) algorithm that performs a stepwise binary splitting using subparameters that maximize the information gain at each decision node. The stopping criteria for splitting were splitting exceeds 5 levels, or the number of samples in the final node is less than 10% of the total (n < 10,000). Finally, the bootstrap aggregating (bagging) method was used to select the most frequent tree structure by performing a majority vote on 10 results^37^. The performance of the classifiers was externally validated using a balanced test dataset and presented as the accuracy of decision nodes and positive predictive values of leaf nodes.

$${\rm{Accuracy}}\,( \% )=({\rm{true}}\,{\rm{positive}}+{\rm{true}}\,\mathrm{negative})/{\rm{total}}\times 100$$

$${\rm{Positive}}\,{\rm{predictive}}\,{\rm{value}}\,( \% )={\rm{true}}\,{\rm{positive}}/({\rm{true}}\,{\rm{positive}}+{\rm{false}}\,\mathrm{postive})\times 100$$

### Estimation of regression coefficients

The entire training dataset was divided into different BIS ranges using the classifiers found in the decision tree analysis. Multiple linear regression analysis was performed for each BIS range, with the EEG subparameters selected in the decision tree as the input and the BIS value as the output. To avoid errors due to outliers, a RANdom SAmple Consensus (RANSAC) regression was performed with a setting of 1,000 samples, 100 iterations, and an outlier margin of 5. The results of the regression analysis are presented as regression coefficients of EEG subparameters and intercepts for each BIS range. The model performance was evaluated by the median error and the median absolute error between the measured and the model-predicted BIS values at each BIS range.

All analyses were performed with the program developed by the authors using scikit-learn (version 0.19.1, https://scikit-learn.org accessed Feb 21, 2018) and SciPy (version 0.14.0, https://scipy.org accessed Feb 21, 2018) libraries and Python Programming Language (version 3.5.2, Python Software Foundation, http://python.org, accessed Feb 21, 2018).

## Supplementary information

Supplementary figure 1
