# Supplementary material for: Data Driven Investigation of Bispectral Index Algorithm
Source: Sci Rep. 2019 Sep 24;9:13769. doi: 10.1038/s41598-019-50391-x (PMC6760206; doi:10.1038/s41598-019-50391-x)

Title: Data Driven Investigation of Bispectral Index Algorithm

Autohrs: Hyung-Chul Lee, Ho-Geol Ryu, Yoonsang Park, Soo Bin Yoon, Seong Mi Yang, Hye-Won Oh, Chul-Woo Jung

Affiliation: Department of Anesthesiology and Pain Medicine, Seoul National University College of Medicine, Seoul National University Hospital, Seoul, Republic of Korea

***Corresponding author E-mail: jungcwoo@gmail.com**

Supplementary figure 1. Time-series changes in subparameters and bispectral index values for 100 randomly selected cases

Blue = observed bispectral index (BIS)	Green = predicted BIS	Red = electromyography amplitude (EMG, dB)
Orange = 95% spectral edge frequency (SEF, Hz)	Pink = relative beta ratio (RBR, unitless)	Gray = burst suppression ratio (BSR, %)

Random case number

1

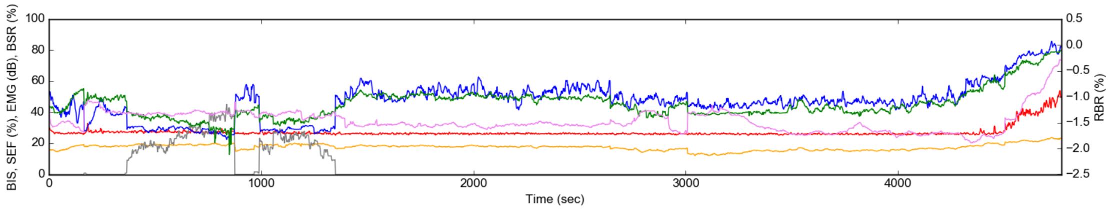

2

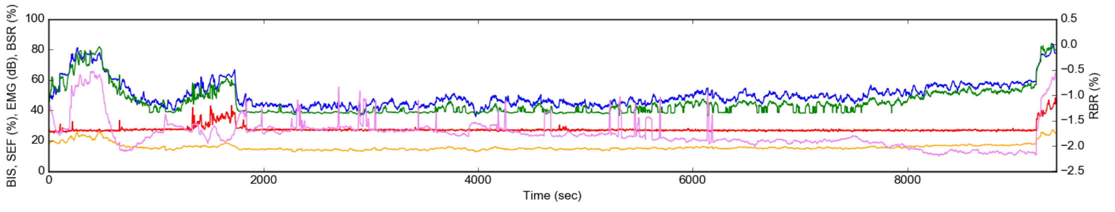

3

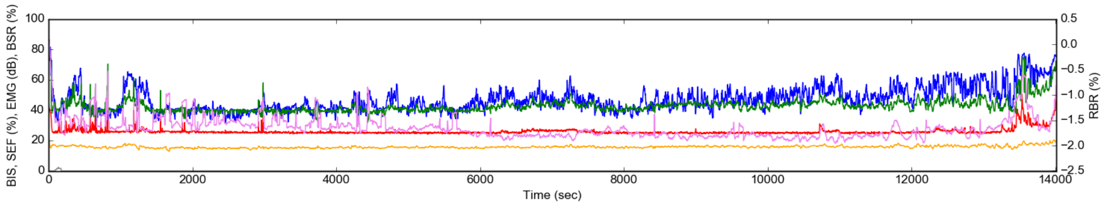

4

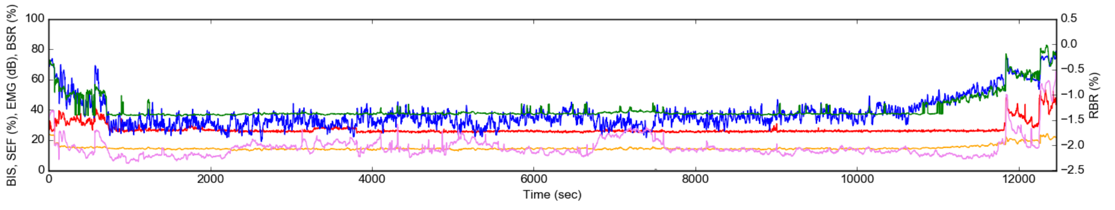

5

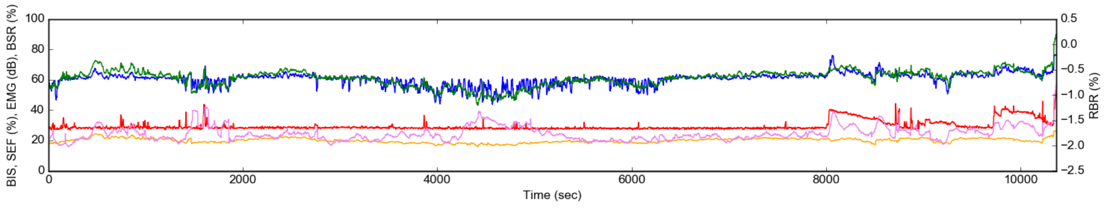

6

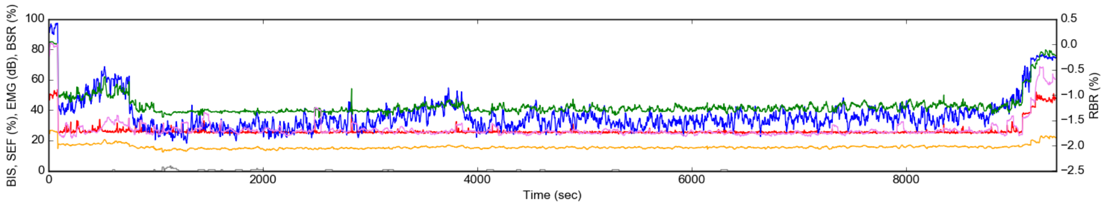

7

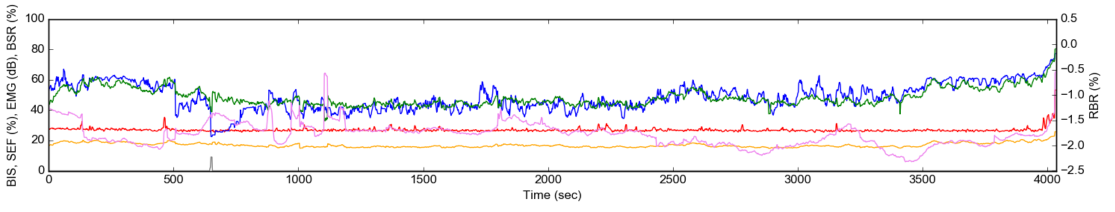

8

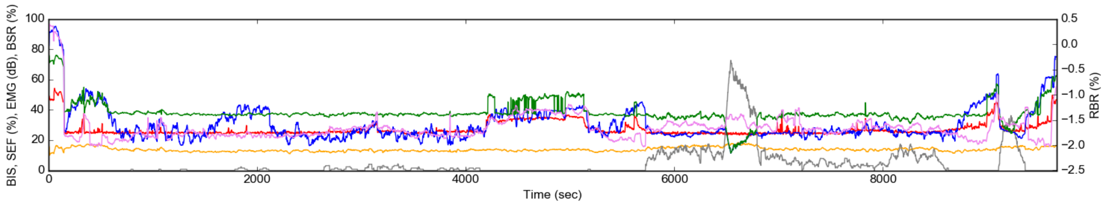

9

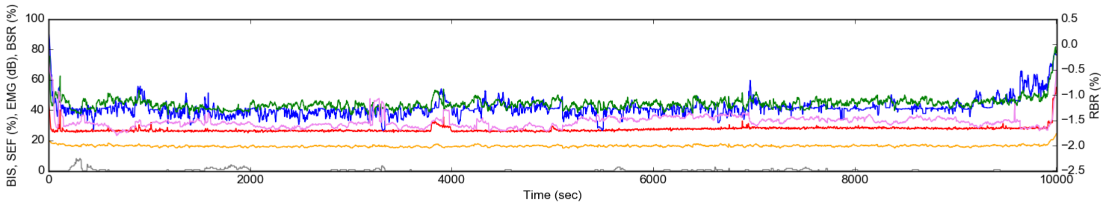

10

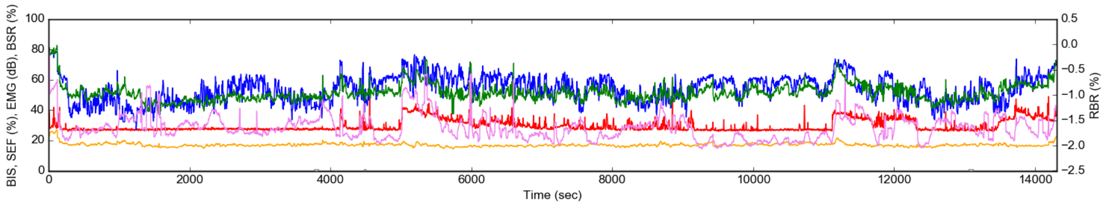

11

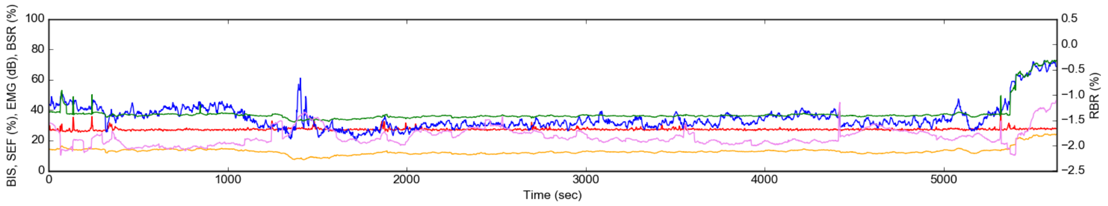

12

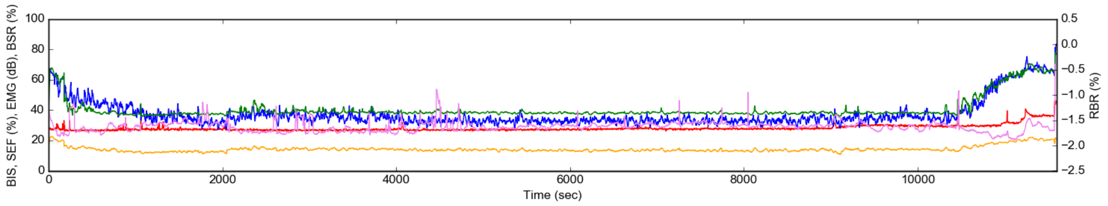

13

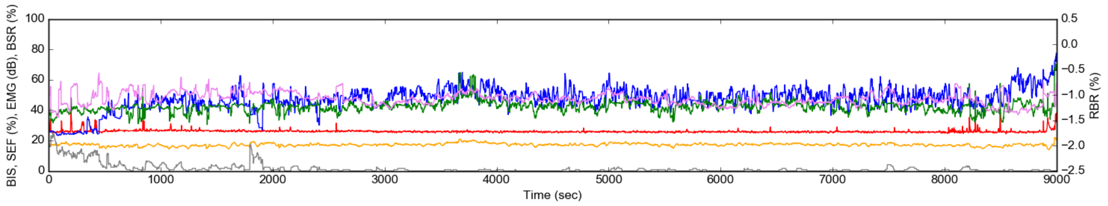

14

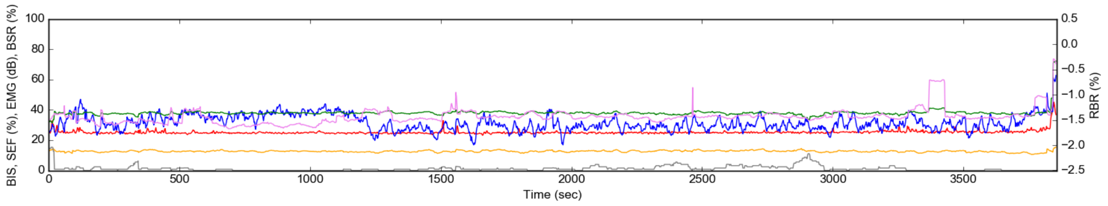

15

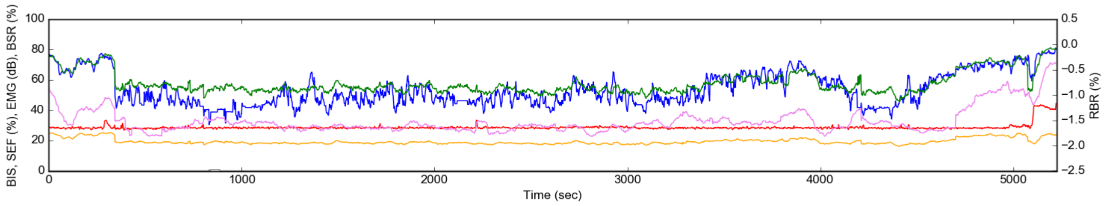

16

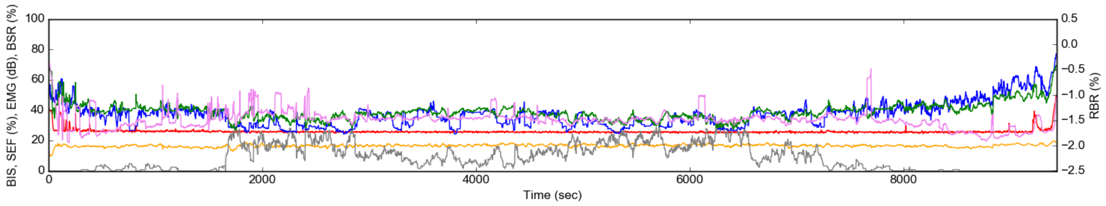

17

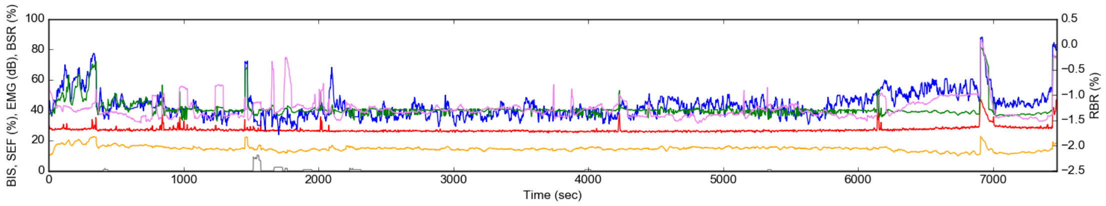

18

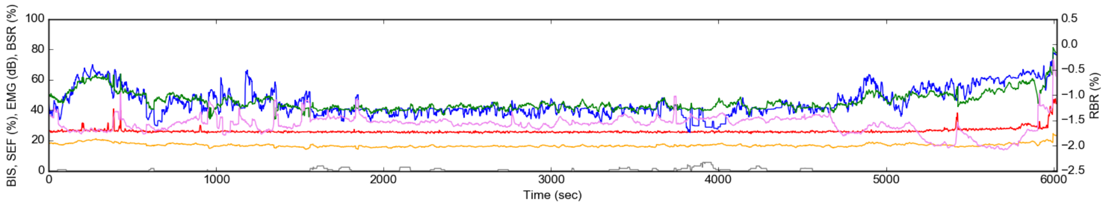

19

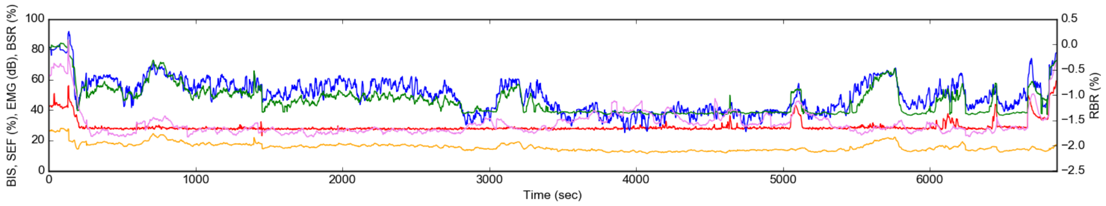

20

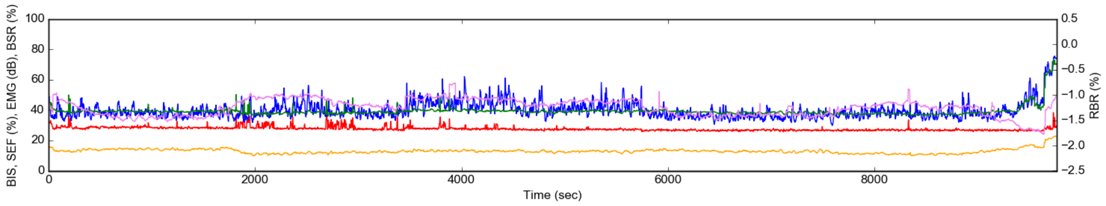

21

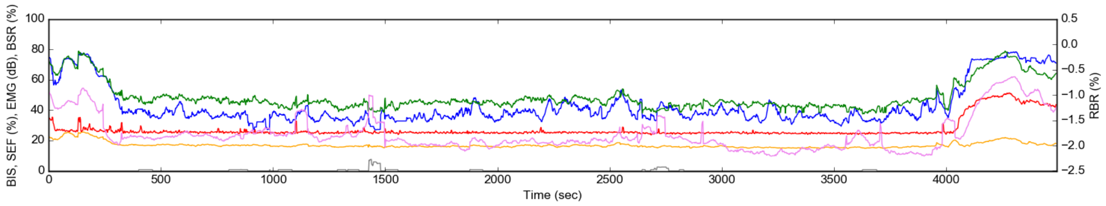

22

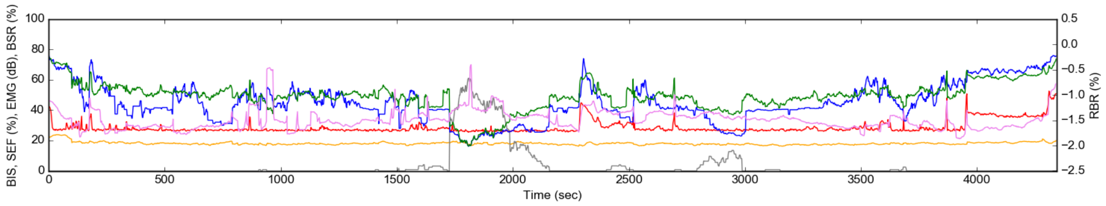

23

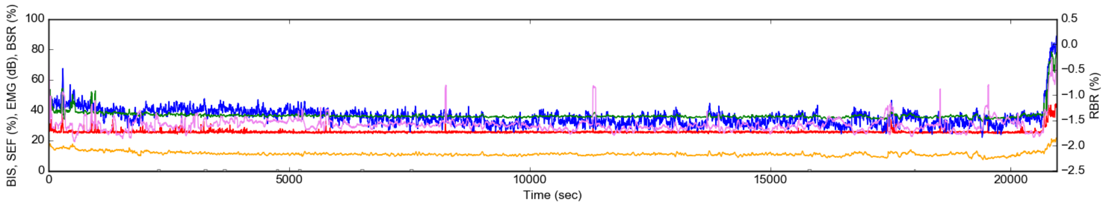

24

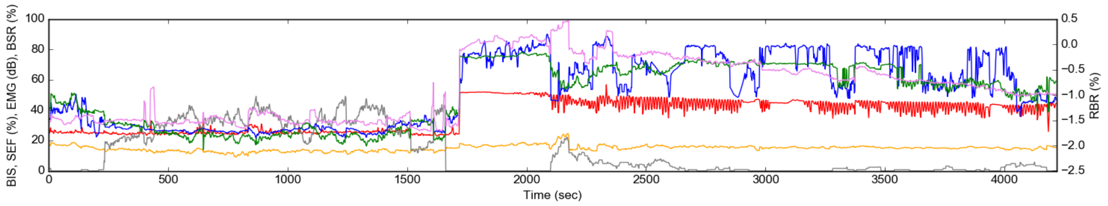

25

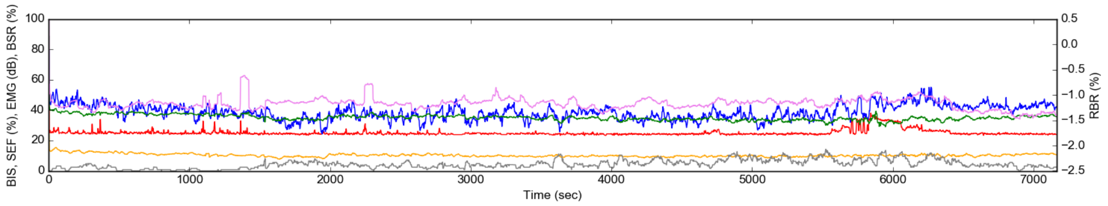

26

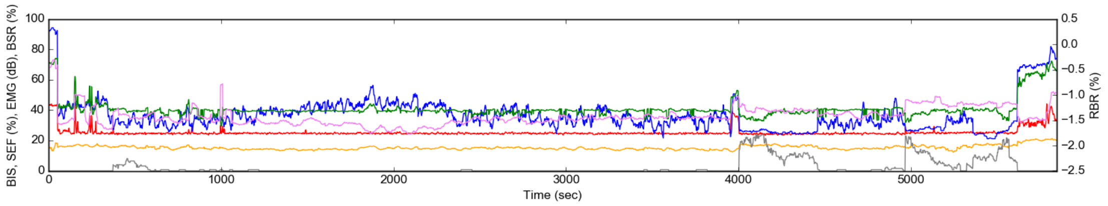

27

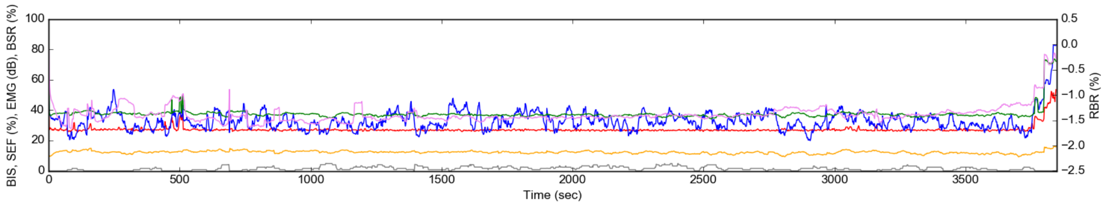

28

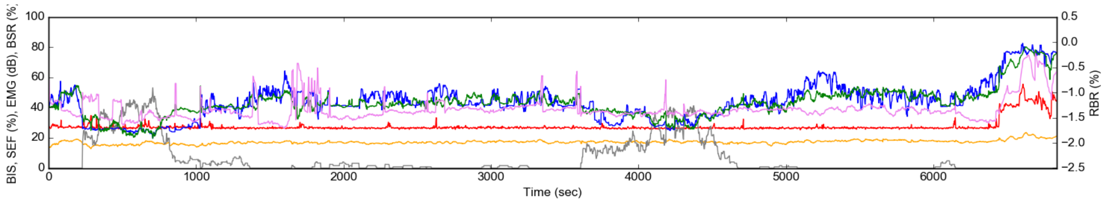

29

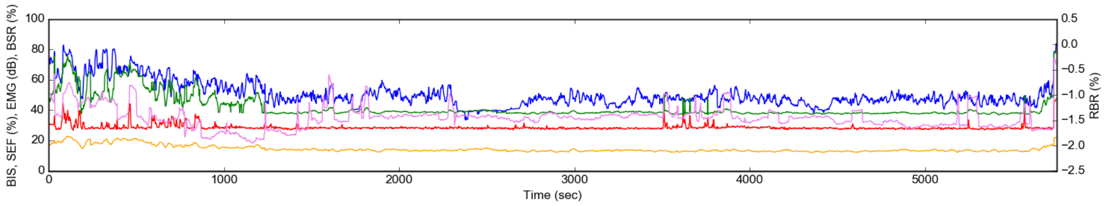

30

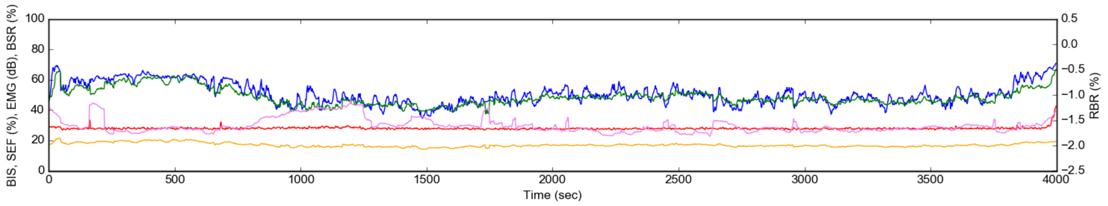

31

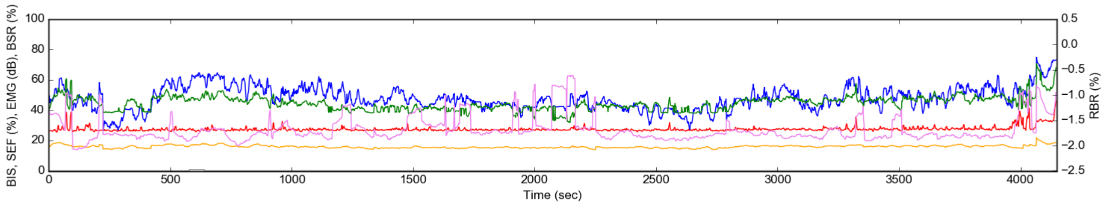

32

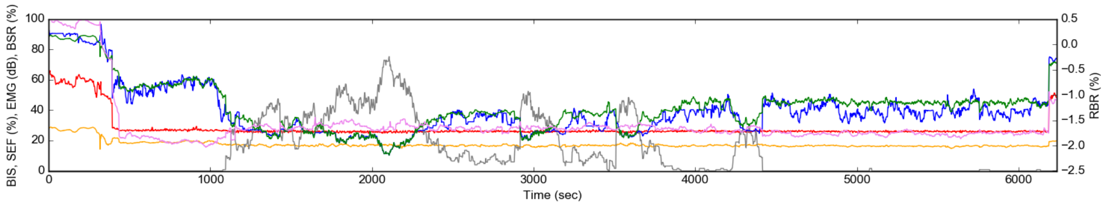

33

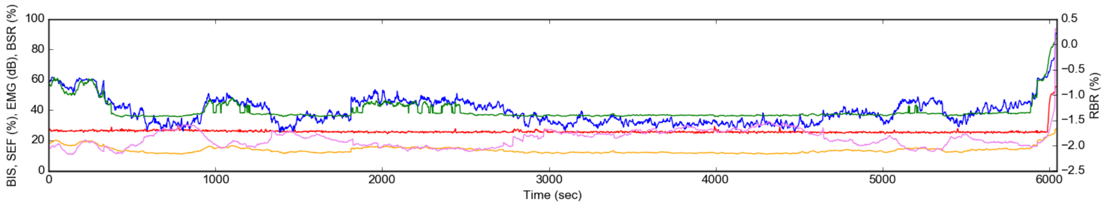

34

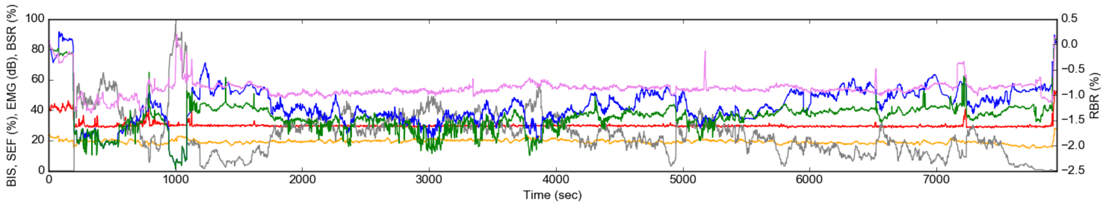

35

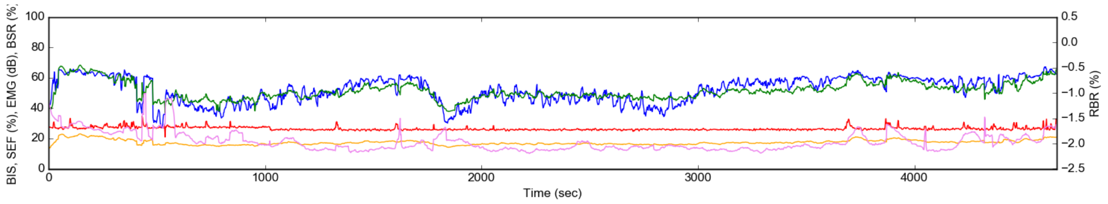

36

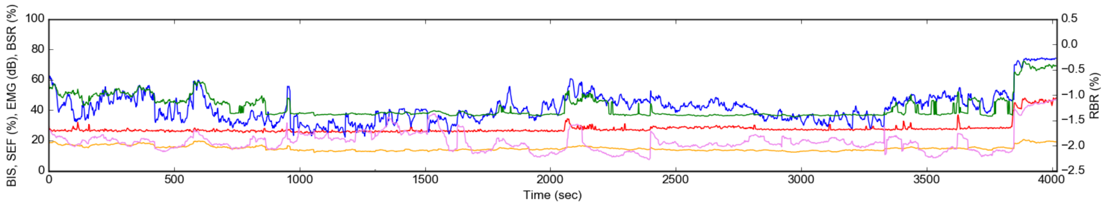

37

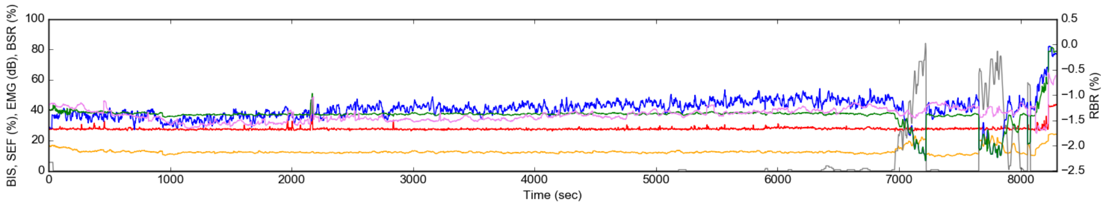

38

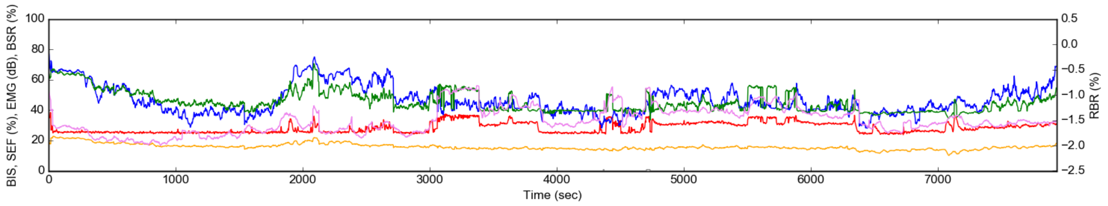

39

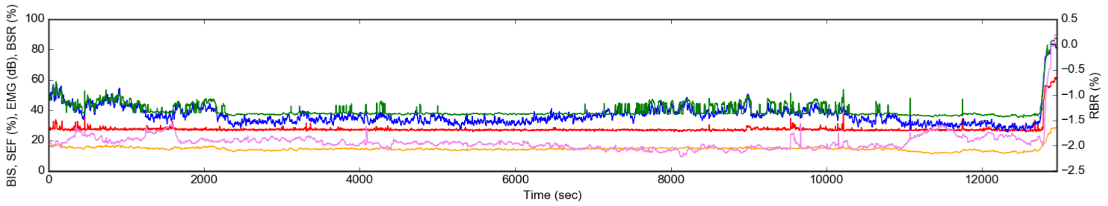

40

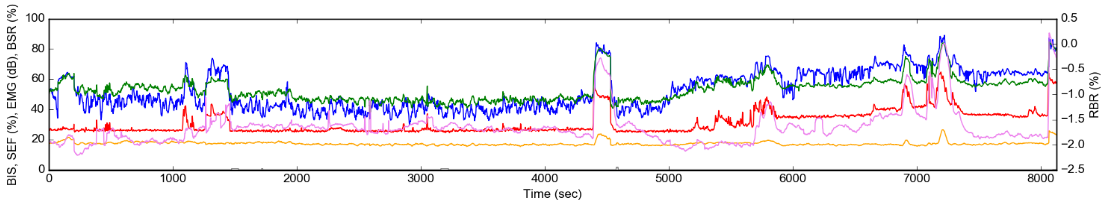

41

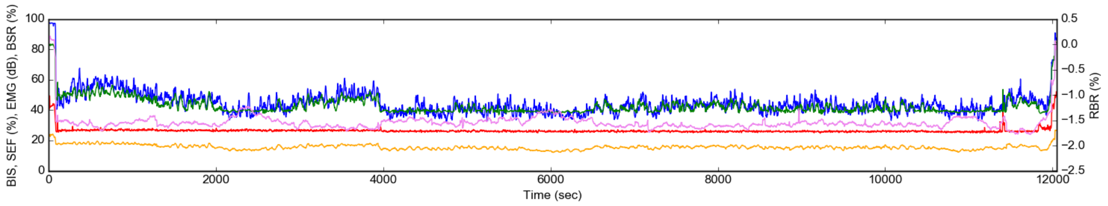

42

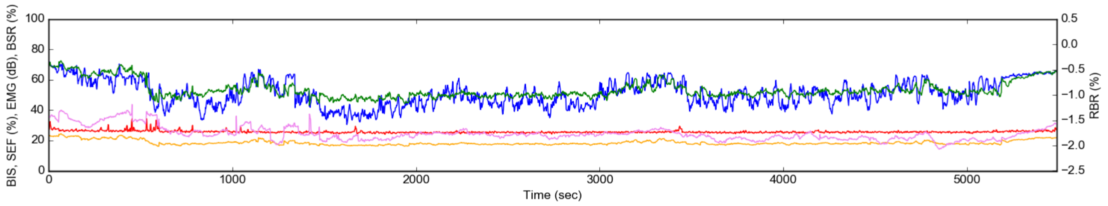

43

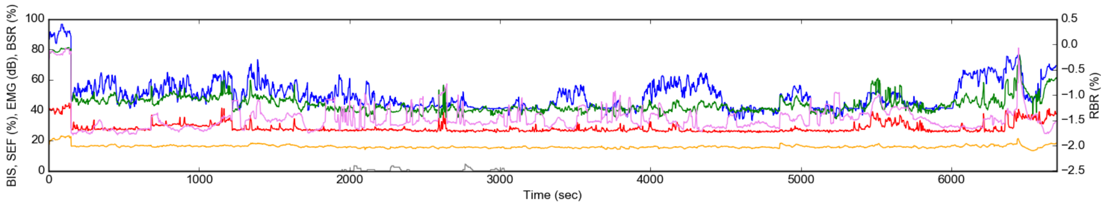

44

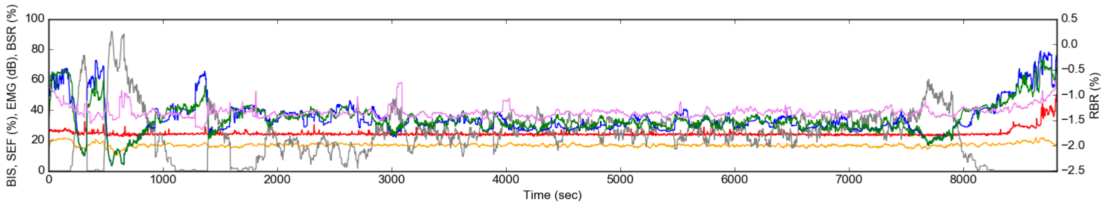

45

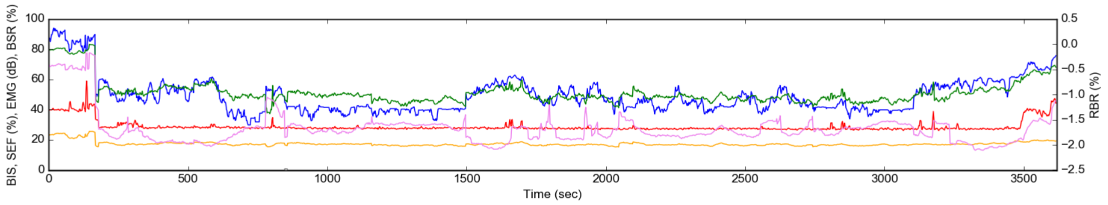

46

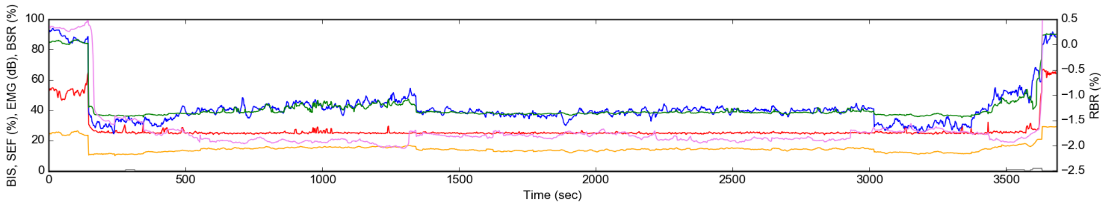

47

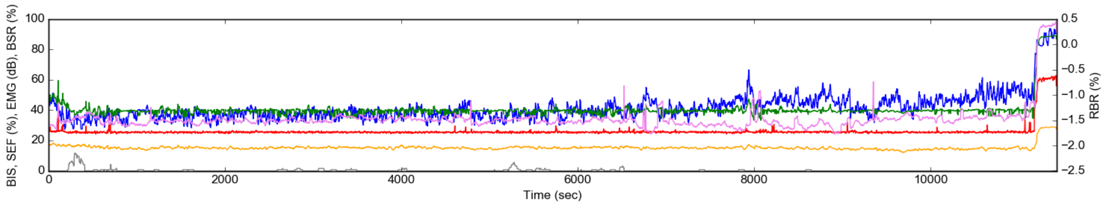

48

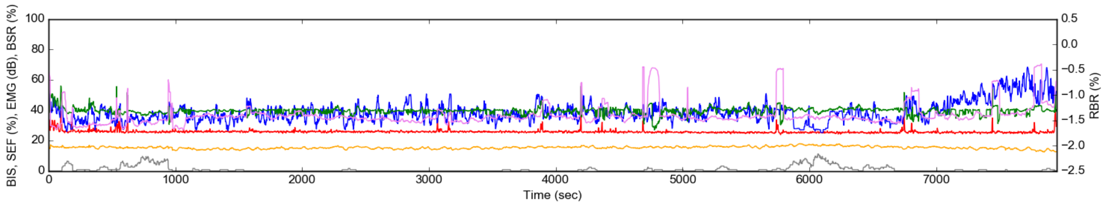

49

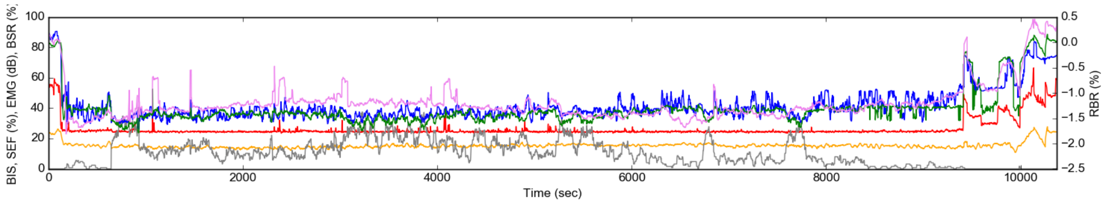

50

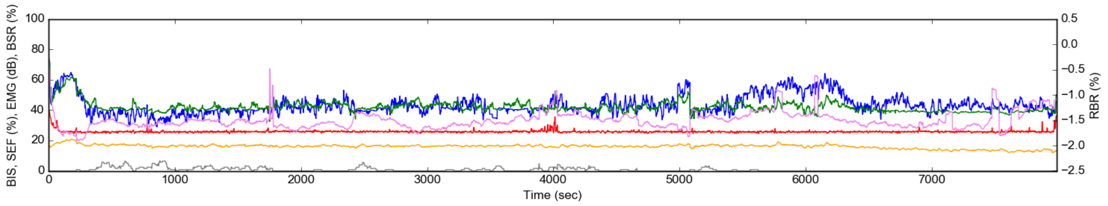

51

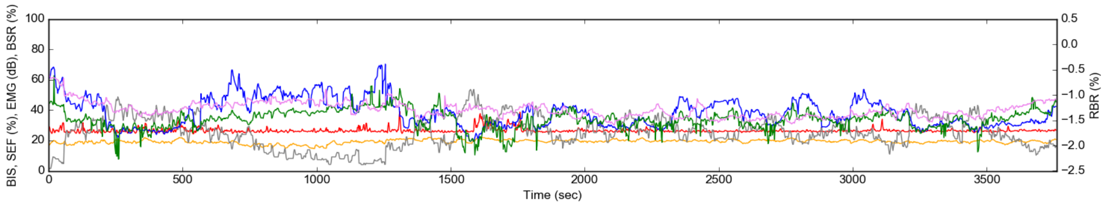

52

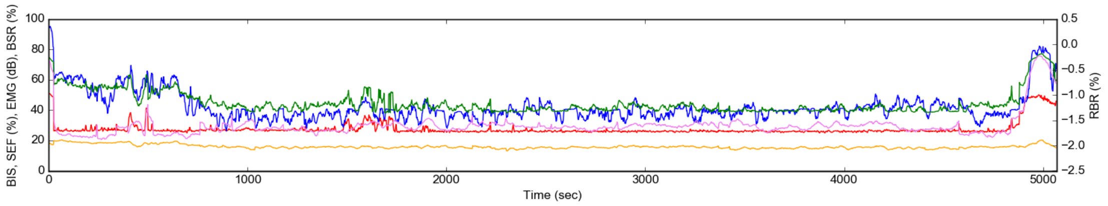

53

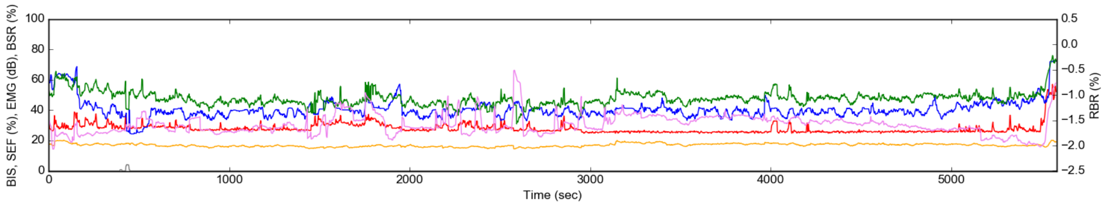

54

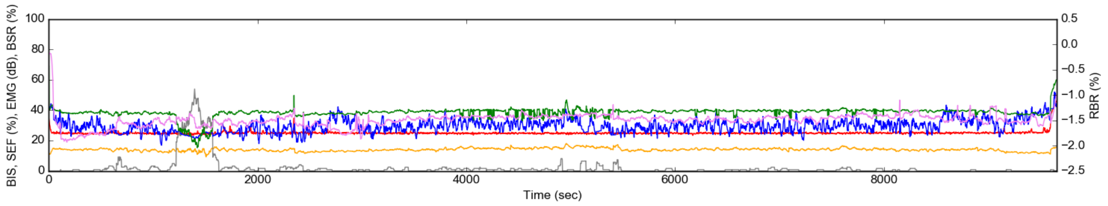

55

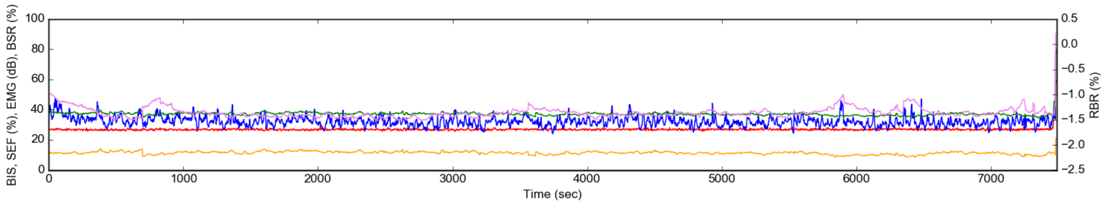

56

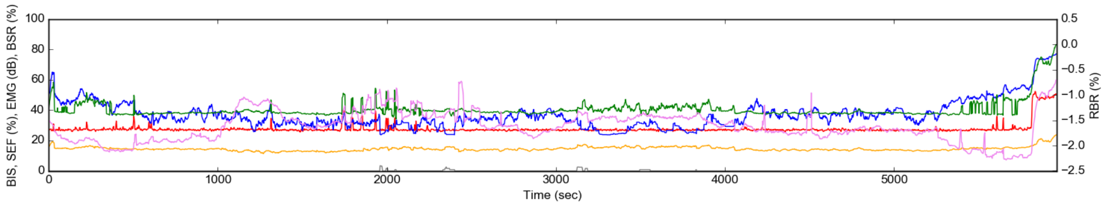

57

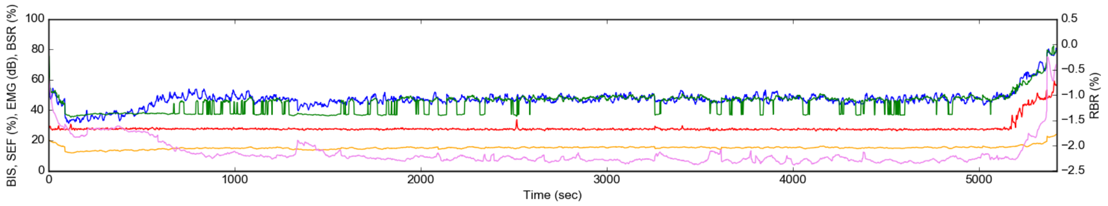

58

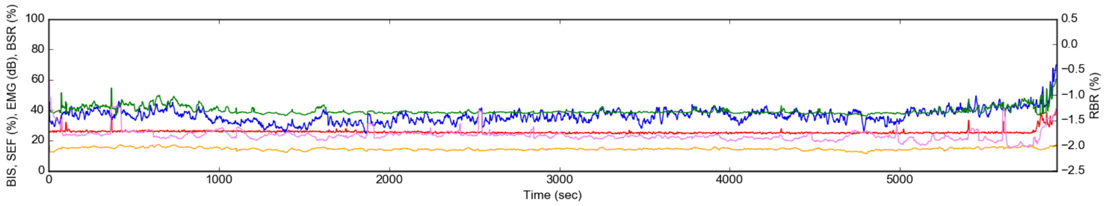

59

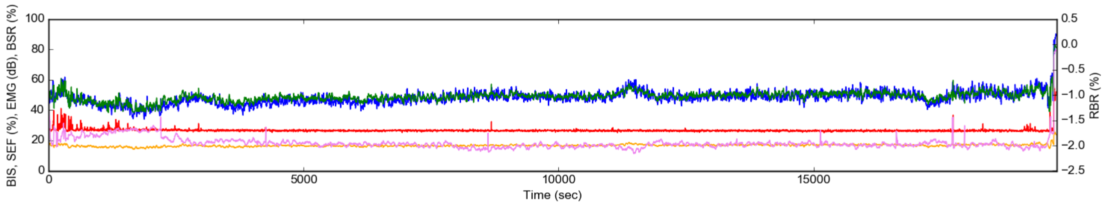

60

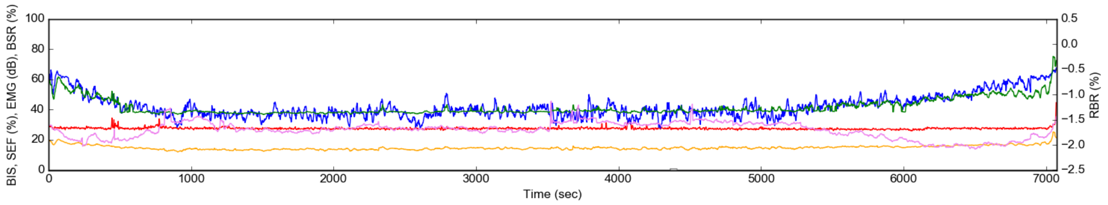

61

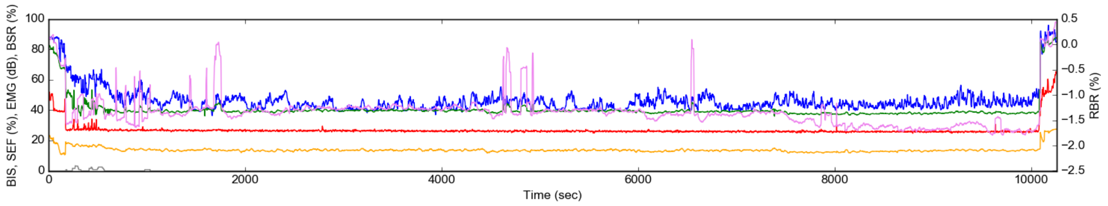

62

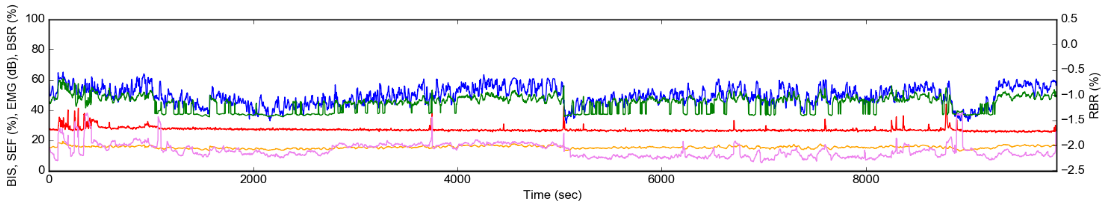

63

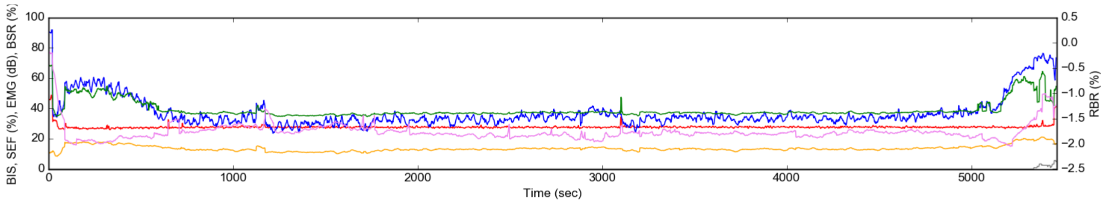

64

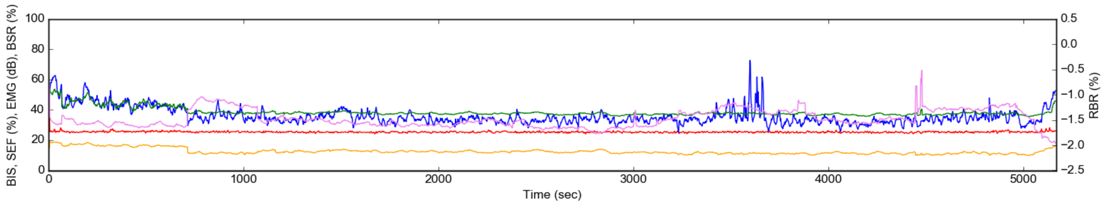

65

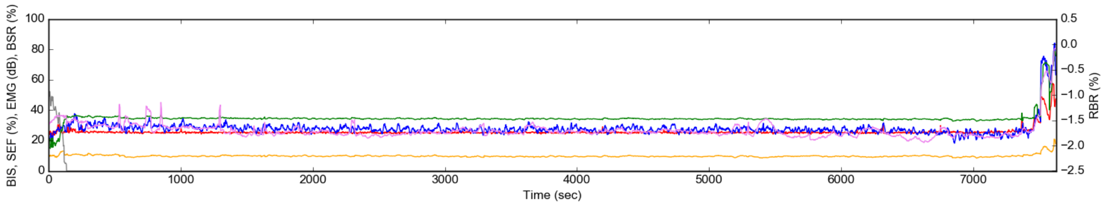

66

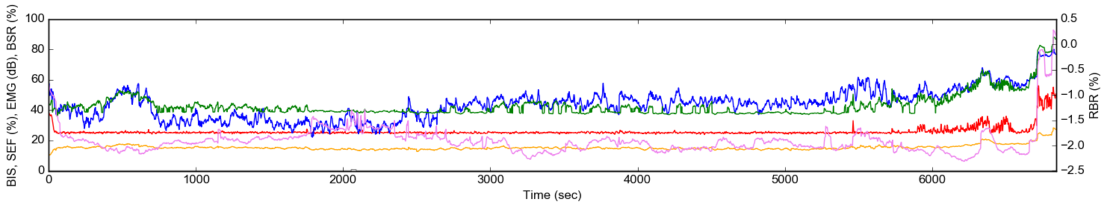

67

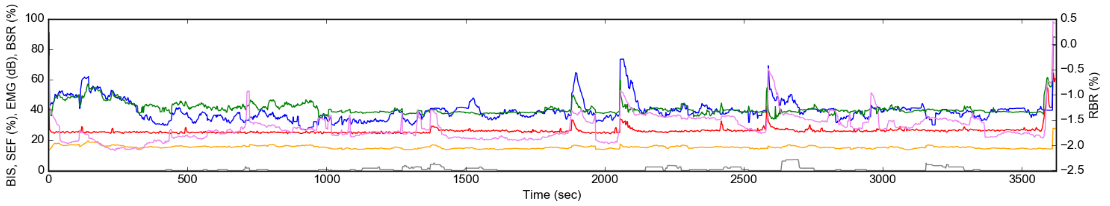

68

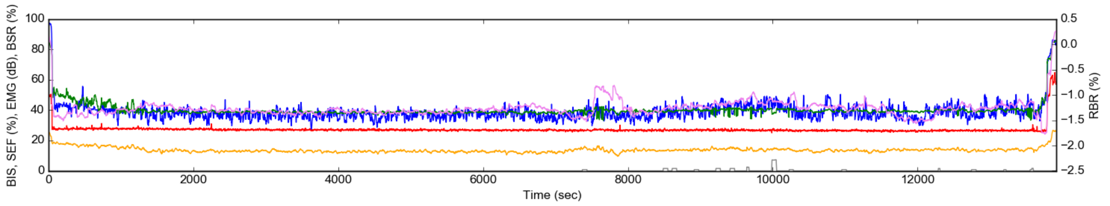

69

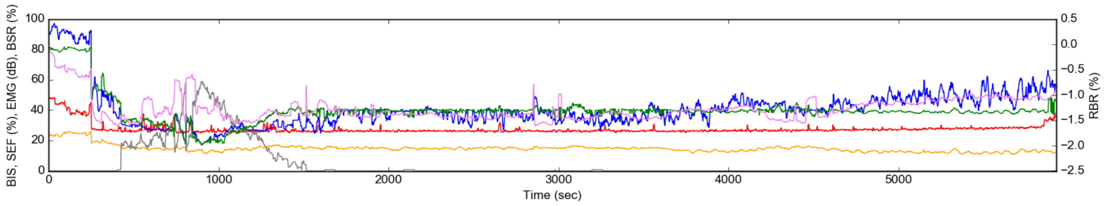

70

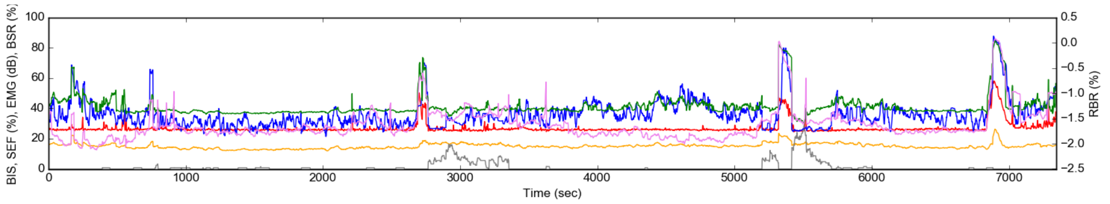

71

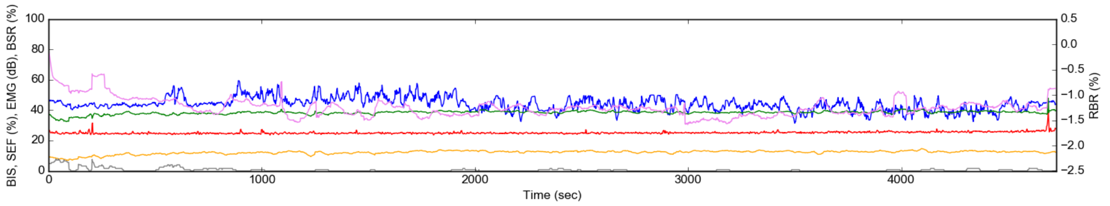

72

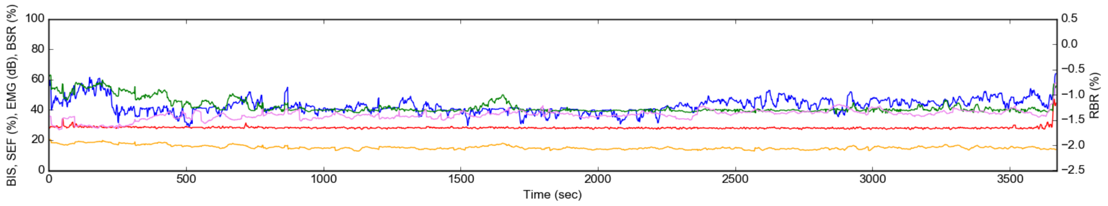

73

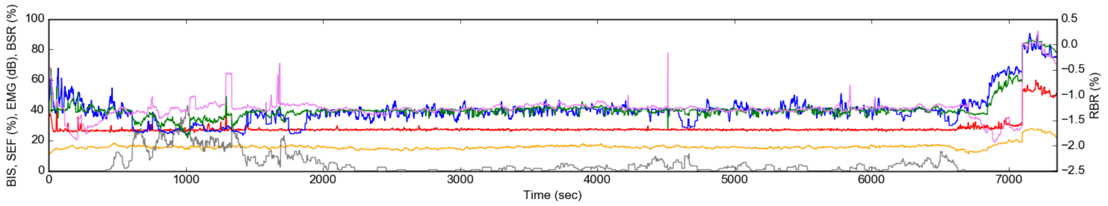

74

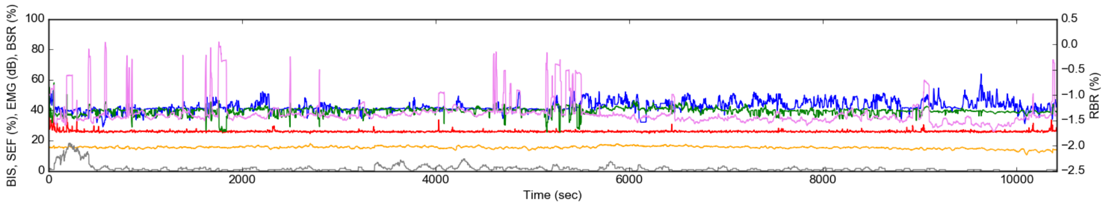

75

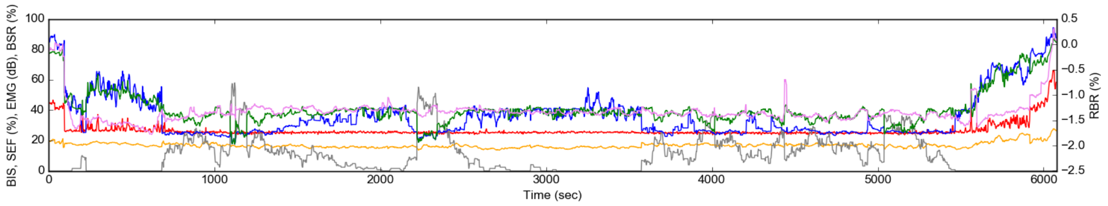

76

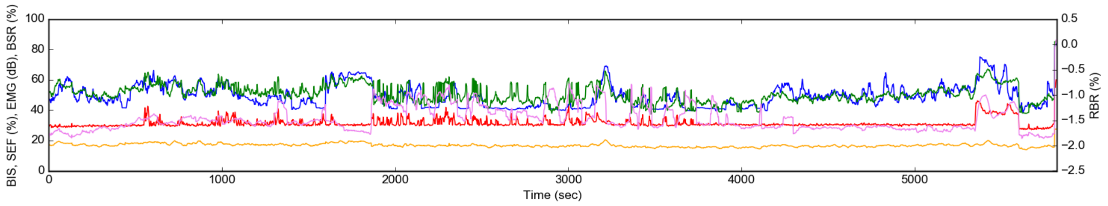

77

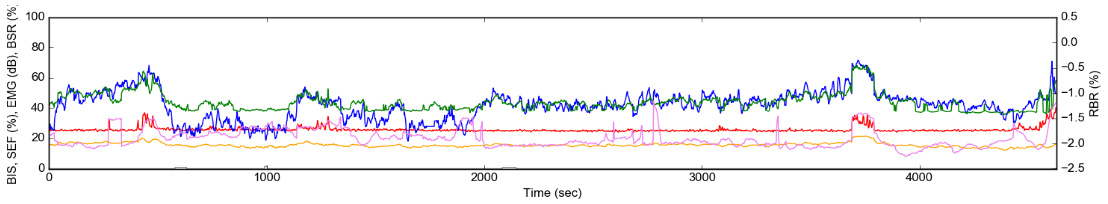

78

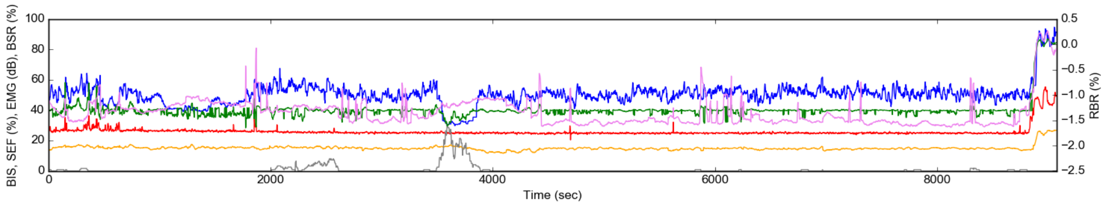

79

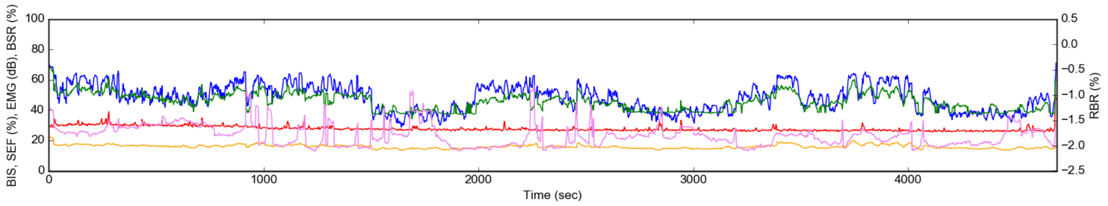

80

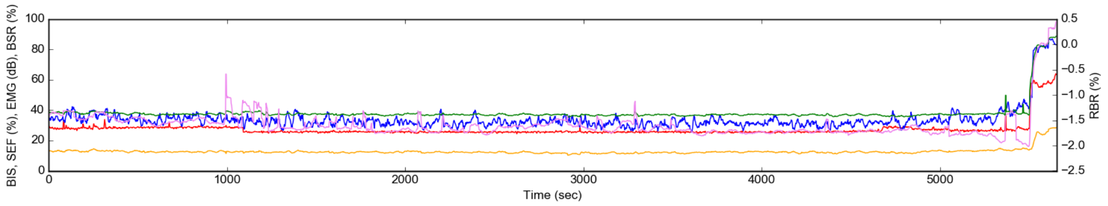

81

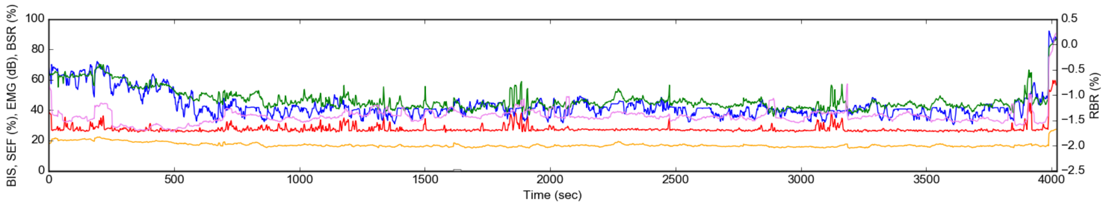

82

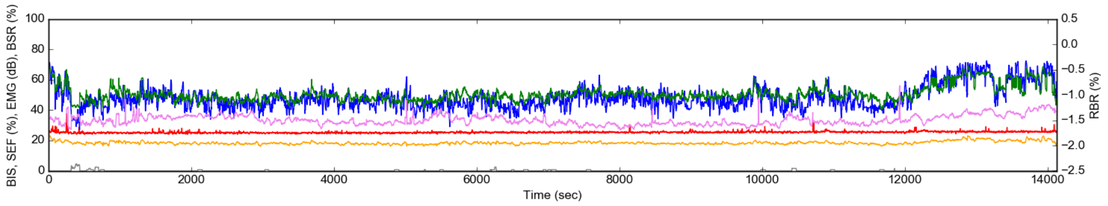

83

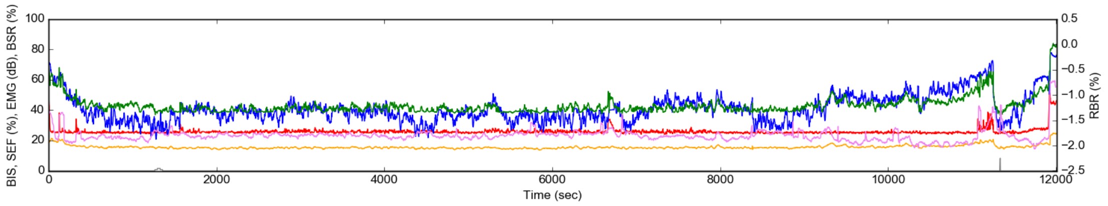

84

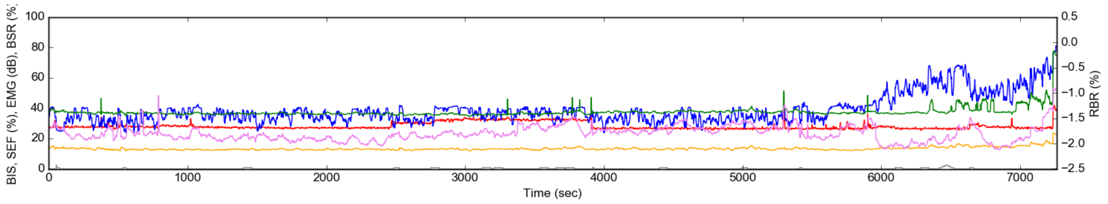

85

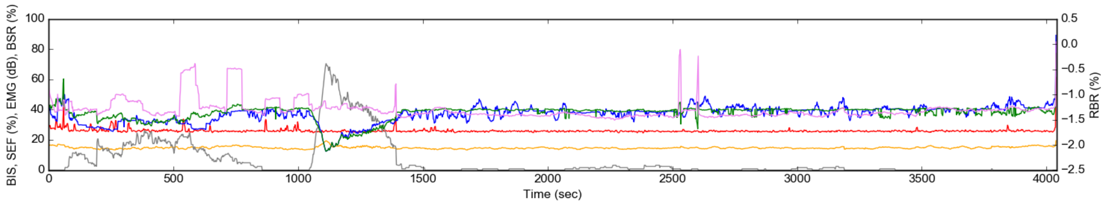

86

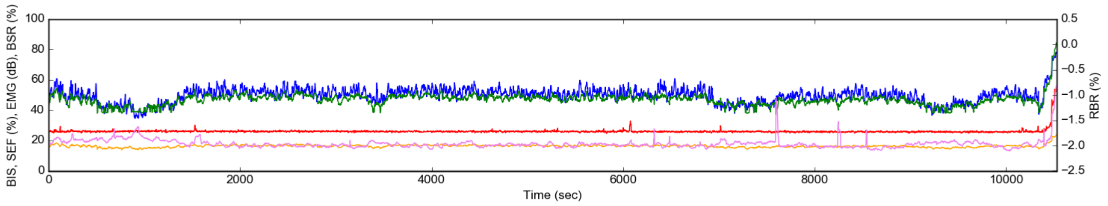

87

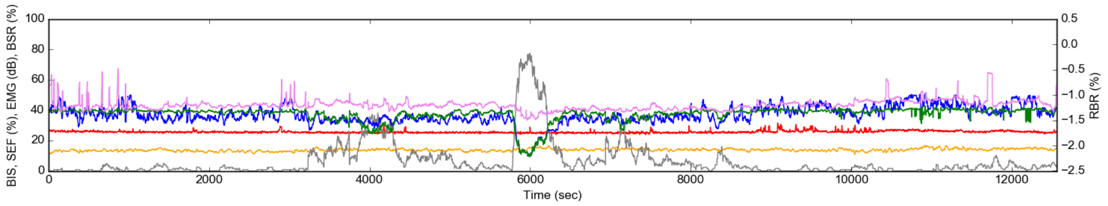

88

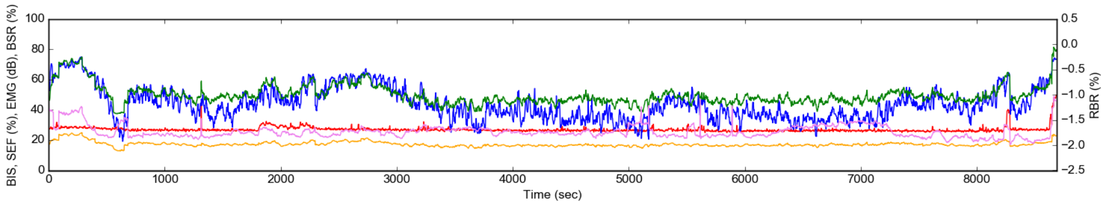

89

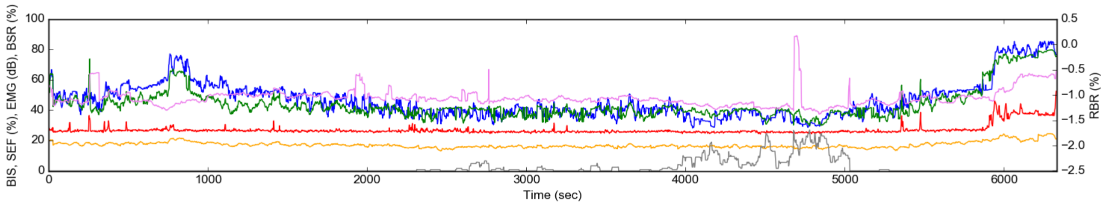

90

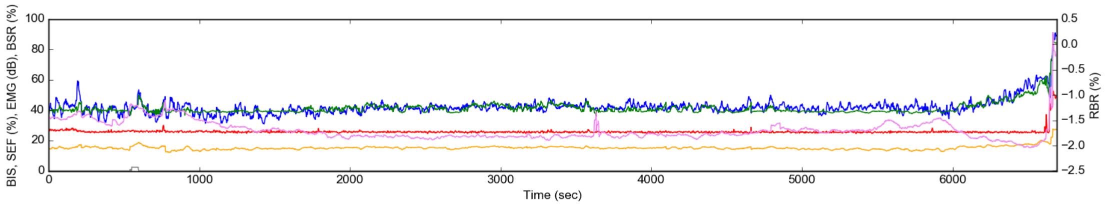

91

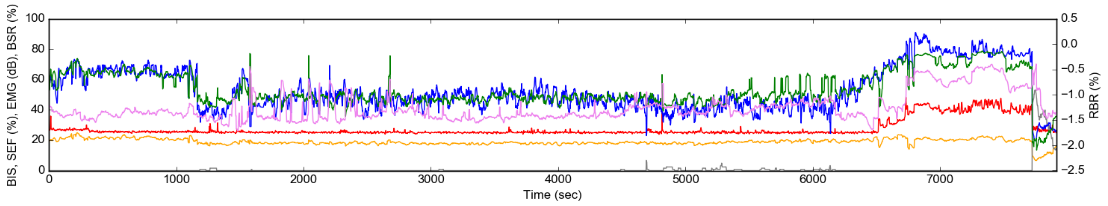

92

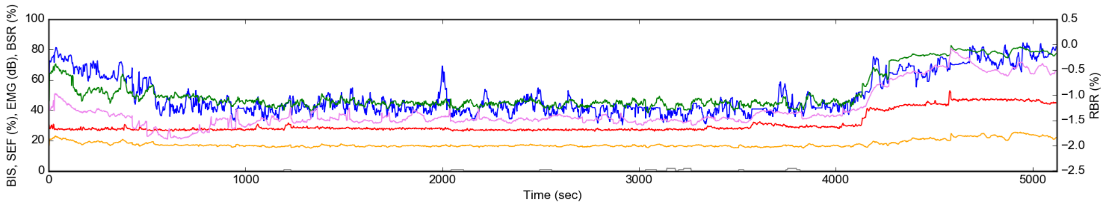

93

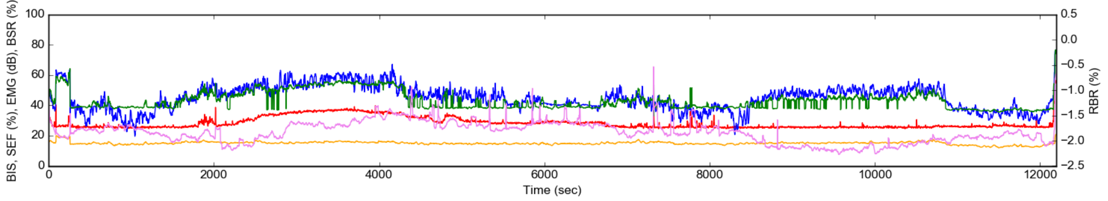

94

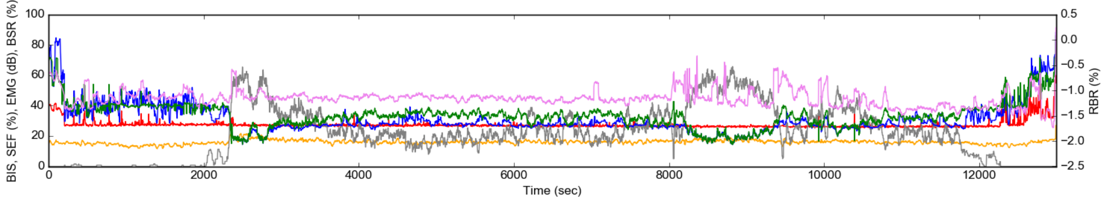

95

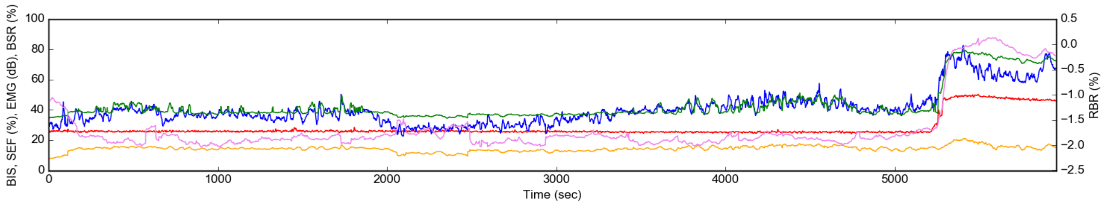

96

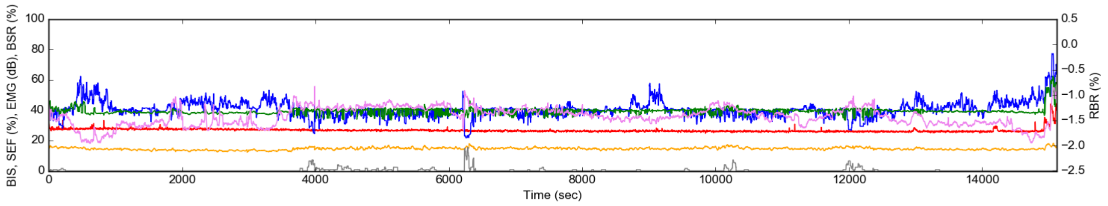

97

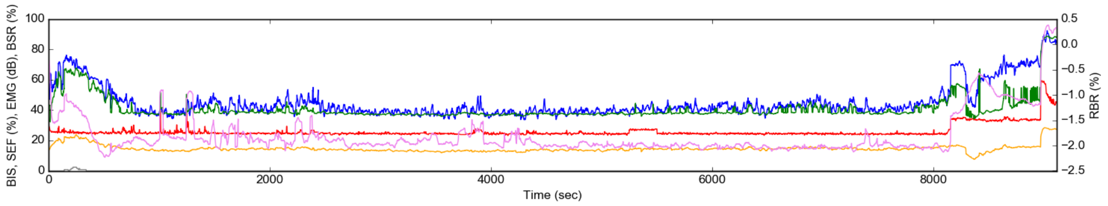

98

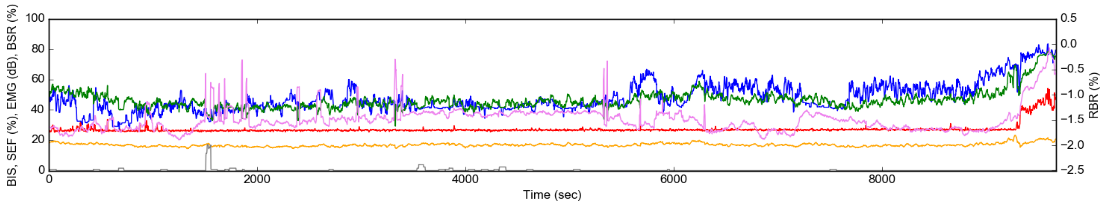

99

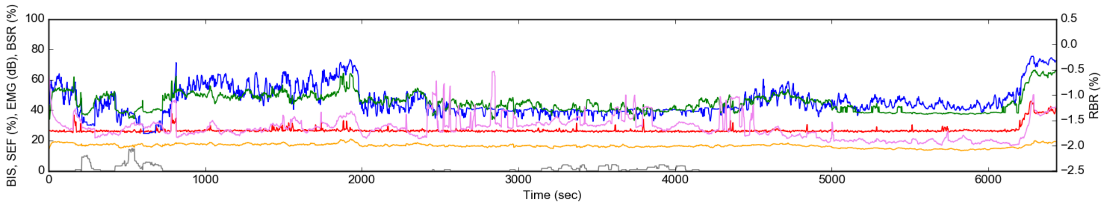

100

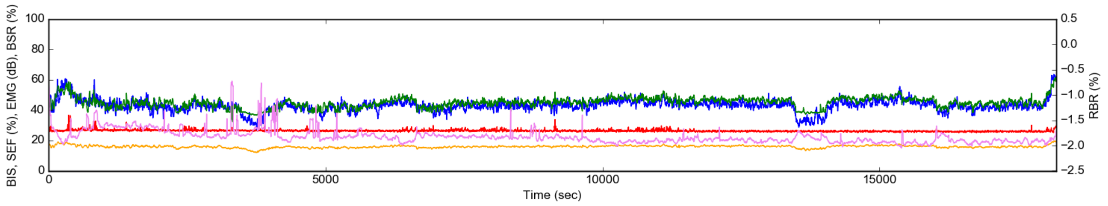

Supplement: Supplementary file 1 — Supplementary figure 1 [file 41598_2019_50391_MOESM1_ESM.pdf]
